# Modeling of Nonlinear Optical Phenomena in Host-Guest Systems Using Bond Fluctuation Monte Carlo Model: A Review

**DOI:** 10.3390/ma14061454

**Published:** 2021-03-16

**Authors:** Antoni C. Mitus, Marina Saphiannikova, Wojciech Radosz, Vladimir Toshchevikov, Grzegorz Pawlik

**Affiliations:** 1 Department of Theoretical Physics, Wroclaw University of Science and Technology, 50-370 Wroclaw, Poland; Antoni.Mitus@pwr.edu.pl (A.C.M.); wojtekradosz@wp.pl (W.R.); 2 Leibniz-Institut für Polymerforschung Dresden e.V., Hohe Strasse 6, 01069 Dresden, Germany; grenzer@ipfdd.de; 3 Institute of Macromolecular Compounds, Russian Academy of Sciences, Bolshoi Prospect 31, 199004 Saint Petersburg, Russia; toshchevikov@imc.macro.ru

**Keywords:** bond-fluctuation Monte Carlo, host-guest systems, photoinduced mass transport, complex dynamics, poling, diffraction efficiency, second harmonic generation, azo-polymers

## Abstract

We review the results of Monte Carlo studies of chosen nonlinear optical effects in host-guest systems, using methods based on the bond-fluctuation model (BFM) for a polymer matrix. In particular, we simulate the inscription of various types of diffraction gratings in degenerate two wave mixing (DTWM) experiments (surface relief gratings (SRG), gratings in polymers doped with azo-dye molecules and gratings in biopolymers), poling effects (electric field poling of dipolar molecules and all-optical poling) and photomechanical effect. All these processes are characterized in terms of parameters measured in experiments, such as diffraction efficiency, nonlinear susceptibilities, density profiles or loading parameters. Local free volume in the BFM matrix, characterized by probabilistic distributions and correlation functions, displays a complex *mosaic-like* structure of scale-free clusters, which are thought to be responsible for heterogeneous dynamics of nonlinear optical processes. The photoinduced dynamics of single azopolymer chains, studied in two and three dimensions, displays complex sub-diffusive, diffusive and super-diffusive dynamical regimes. A directly related mathematical model of SRG inscription, based on the continuous time random walk (CTRW) formalism, is formulated and studied. Theoretical part of the review is devoted to the justification of the a priori assumptions made in the BFM modeling of photoinduced motion of the azo-polymer chains.

## 1. Introduction

### 1.1. Outline

Host-guest systems play an important role in studies and applications of nonlinear optical effects [1]. Those systems consist of various types of guest molecules and particles, such as the dipolar and octupolar molecules, coated spheres, photo-isomerizable molecules, etc. dispersed in a host matrix. The latter can be a liquid crystal, polymer, biopolymer or other material. The nature of interactions between the guest and host components varies from case to case. In nonlinear optical applications the host-guest systems interact with the electromagnetic field via various types of light-matter interactions, resulting in a multitude of physical effects [1].

Theoretical studies of nonlinear effects, based on quantum-mechanical concept, offer a deep understanding and satisfactory description of a large variety of experimentally observed effects [2,3]. Those studies concentrate mainly on physical effects directly related to the guest component, i.e., embedded molecules.

Theoretical study of host-guest systems constitutes a challenge. Its origin lies in the fact that polymer systems exhibit an intrinsically hierarchical response on many length and time scales [4] and belong, in general, to the class of systems with complex heterogeneous dynamics. An analysis of static and dynamic physical phenomena in those systems requires a detailed characterization of typical configurations of polymer chains affected by the guest molecules on microscopic and mesoscopic scales. Statistical physics of polymer systems on mesoscopic scales concentrates on different aspects of their static and dynamic properties [4,5,6,7,8]. Characterization of typical local structure of polymers, as well as its counterpart for simple liquids [9], constitutes, in our opinion, one of most important unsolved problems of equilibrium statistical mechanics of classical systems.

Computer simulations provide complementary tools for the study of physical processes on various temporal and spatial scales. In particular, the molecular dynamics (MD) method, the Brownian dynamics (BD) method as well as the Monte Carlo (MC) method have been used to simulate the mesoscopic molecular models in polymer physics [10,11,12], opening the possibility of numerical studies of host-guest systems. In this paper we review the results of modeling of chosen nonlinear optical effects in host-guest systems, using one of the efficient simulation techniques—MC Bond Fluctuation Method (BFM).

### 1.2. Monte Carlo Simulations of Polymer Systems

Various simulation techniques are used to model the polymer systems, depending on the length and time scales [10,13], as well as on the details of the characterization of the system. For problems involving atomistic features the quantum chemical calculations are used [14], which provide detailed information on the scale of, say, a single polymer chain. On larger scales, when tens or hundreds of polymer chains are of interest, molecular dynamics (MD) methods are used—the polymer chain dynamics is simulated by numerically solving Newton’s equations, using either model potentials or potentials obtained from quantum-mechanical simulations. In an alternative approach the Langevin’s equation is numerically integrated. For the class of problems for which still much larger polymer systems (consisting of, say, $10^{4}$–$10^{5}$ chains) are necessary, those methods become insufficient and simplified models are used. For polymer chains this approach requires replacing successive chemical groups by an effective bond between some effective units. The MC method, discussed in detail in Section 2.3.1, offers the possibility of generating a set of instantaneous configurations for systems consisting of a large number of model physical objects like, e.g., model polymer chains, at the price of being less detail-specific than its quantum-mechanical or molecular dynamics counterparts. In the case of non-linear optics experiments in host-guest systems the effects typical for complex systems are often observed, indicating that large spatial scales play an important role. Consequently, Monte Carlo methods are adequate for their theoretical modeling.

Monte Carlo simulations use off-lattice as well as lattice models. Off-lattice method models a polymer chain as consisting of rigid bonds of a constant length, jointed together at arbitrary angles, or as a bead-spring system [4,6]. In the latter case various interactions between all beads [15] and/or a potential controlling the angle between subsequent bonds are introduced.

In lattice models each effective bead of the polymer occupies a single lattice site; the nearest-neighbor beads are connected by links. These links simulate the Kuhn segments formed from groups of some number of monomers along the polymer chain: averaged length of the links in the BFM equals the length of the Kuhn segment of the polymer chain. The assigned potential energy depends on the length of the link. One of the commonly used algorithms for the simulation of many-chain systems is the BFM [16,17,18]. It combines typical advantages of lattice MC methods with those from the continuous BD algorithm. The details of BFM are discussed in Section 2.3.2.

In spite of being a non-specific model of polymer dynamics, the BFM was successfully used to study a large variety of physical effects in two- and three dimensions like, e.g., static [18,19,20,21,22,23,24,25,26,27,28,29] and dynamic [18,30,31,32,33] properties of linear chains, polymer rings [27,34], polymer blends and interfaces [35,36], gels and networks [37], glass transition [38,39,40], polymer blends [41], (co)polymers at surfaces [42], polymer brushes in good solvents [43,44,45,46,47,48,49], polymer thin films [50,51,52], equilibrium polymers [29,53,54], general self-assembly [55,56,57], networks and gel point [58,59,60,61], olympic gels [62,63,64], hyperbranched polymers [65,66], dendrimers [67,68,69,70], lipid membranes [71,72,73,74], see also review papers [75,76,77].

### 1.3. Photoinduced Mass Transport in Functionalized Azo-Polymers: Concepts

The modeling of physical effects in azopolymers, i.e., host-guest systems with photo-isomerisable molecules attached to the polymer chain, requires some knowledge about the origin of light-induced mass motion. This topic has, since long time, created some controversies, presented in detail below.

The most distinctive feature of azopolymers is their ability to change a shape and to exhibit sofisticated biomimetic motions under light illumination [78,79,80,81]. For example, the surface of thin azopolymer films easily deforms when irradiated by the polarized visible light and the deformations can be directed by the light polarization [82,83]. The nature of the physical mechanism behind surface relief changes stays still a debatable issue, if judged by a growing number of theories presented for its explanation [84,85,86,87,88,89,90,91,92,93]. Most theoretical developments rely on the concept of photo-induced plasticization [85] or directional photo-fluidization [94,95]. However, a number of experiments carried both by the protagonists [94,96,97] and antagonists [98,99,100,101] of the photofluidization concept failed to provide unambiguous evidence of macroscopic light-induced fluidization in the glassy azo-polymers. Under illumination with visible light the mechanical moduli decrease at most two-four times and the viscosity of glassy azo-polymers diminishes less than one order of magnitude at conventional laser intensities [102]. This means that material constants are only slightly changed during the process of azo-isomerization. The azo-polymer stays in the glassy state and does not undergo a photofluidization transition.

By saying this, we should distinguish between two kinds of azo-materials. The first kind is used in nano- and micro-technologies to inscribe surface relief patterns or produce lithographic posts [103,104]. To ensure a long-term thermal stability (ranging from days to months), one chooses such materials which are deep in a glassy state. Their glass transition temperature $T_{g}$ is nearly 100 K above the ambient one or in the case of azo-polyurethanes [105] even much higher. On the other hand, in some applications it is desirable to have the materials which can be easily switched by light illumination between the solid and liquid state. First we mention a class of low molecular crystalline azo-materials whose melting temperature can be abruptly lowered by irradiation with ultraviolet light, when all chromophores are being pushed from the trans- to the cis-state. If the cis-crystal of such material has the melting temperature below the ambient one, a spectacular crawling along the surface [106] or even jumping from it have been observed [107]. Second, nowadays a special chemistry design allows to produce the azo-polymers, the glass transition temperature of which can be decreased to the region below the room temperature by illumination with ultraviolet light [108,109,110]. Molecular features of this kind of polymers provide a photoinduced solid-to-liquid transition. In particular, their side-chains contain azobenzene-type chromophores connected with the long alkyl chains as spacers and tails. In contrast, azo-polymers used to inscribe surface relief patterns or produce lithographic posts does not contain long alkyl chains and accordingly no decrease of $T_{g}$ was reported for them, even under irradiation with ultraviolet light [109].

In any case to clarify the directional deformations in glassy azo-polymers, one needs a sound physical concept which can explain a strong photomechanical coupling between the processes taking place in these materials on a microscopic and macroscopic levels. Recently, a strong evidence appeared that the irradiation with polarized visible light is able to generate extremely large stresses [111,112,113] which induce mass transport leading to appearance of surface relief patterns. The origin of such large stresses has been predicted already a decade ago in the frame of orientation approach [102,114], the basic features of which have been checked in coarse-grained modeling [115,116] and recently received a rigorous theoretical proof [117,118]. The orientation approach is feeded by the idea that the reorientation of azobenzene chromophores, known to occur perpendicular to the polarization direction of light, is followed by rearrangement of backbones of macromolecules coupled to them [114,116,119,120]. Importantly, the orientation approach predicts the values of the light-induced stress, which is large enough to deform irreversibly glassy azo-polymers in agreement with experiments [112,113,121]. Theoretical formulation of this topic is given in Section 2.1.

The photoinduced mass transport has been also observed in azo-containing polymer brushes [111] and azobenzene stars [122,123].

### 1.4. Scope of the Review

The original BFM was generalized to account for guest molecules, interacting with external fields: constant electric field and laser light. Three types of organization of host-guest systems are studied, in which the guest molecules are (i) embedded in a polymer matrix, (ii) attached to the polymer chain and (iii) semi-intercalated in the polymer chain. Systems are studied in the weak concentration limit, when the interactions between guest molecules can be neglected.

The review covers three groups of topics. The first one, modeling of chosen non-liner optics effects, was motivated by experimental results. They include, e.g., inscription of various types of diffraction gratings, second harmonic generation (SHG), all-optical poling, light-driven mass transport and others. Typically, the physical problem is formulated and the corresponding BFM model presented, followed by simulation details and physical interpretation of the results. Simulations offer a detailed insight into “microscopic” processes, promoting formulation of a physical picture corresponding to experimental effects and, in some cases, of mathematical models.

A second group has a methodological character and is related to the classification of types of light induced dynamics of azo-polymers. It offers a good starting point for simple mathematical models (like, e.g., continuous time random walk) in which photoinduced mass transport of azo-polymers becomes important. We point out that some aspects of photoinduced motion of azo-polymers were discussed by other groups [86,92,124,125].

Third group of topics offers a partial answer to the challenging problem of a classification of local structure of model polymer system. It is limited to a part relevant to the study of host-guest systems—the statistical characterization of local free volume voids) in a polymer matrix.

The paper is organized as follows. Section 2 is divided in two parts. The first one has a theoretical character and provides an analytical treatment, based on the kinetic equations for photo-isomerization transitions, of the stress in polymeric materials resulting from interaction of the light with photo-switchable molecules. The second part reviews the generalized BFM for host-guest molecules in the presence of external electromagnetic fields and defines the parameters typical for the nonlinear effects discussed in this review. The three groups of topics briefly introduced above are presented in Section 3.

## 2. Materials and Methods

### 2.1. Theory: “Microscopic” Orientation Potential

Description of the photo-induced mass transport in azobenzene-containing polymers is based on the angular-dependent photoisomerization of azobenzene chromophores with respect to the polarization vector of the light. Angular-dependent photoisomerization process leads to the photo-induced orientation ordering in the azo-polymer which according to the stress-optical law [4] should be accompanied by the mechanical stress. Below we discuss first the kinetic equations of photoisomerization under linearly polarized light and then present the generalization of the formalism for the elliptically polarized light.

#### 2.1.1. *Linearly Polarized Light*

The probability of trans-cis isomerisation of azobenzene under linearly polarized light depends on the angle θ formed by the transition moment of the trans-isomer with respect to the polarization vector of the light E. In a good approximation it can be assumed that the transition moment is directed along the long axis of the rodlike trans-isomer. According to Dumont and Osman [126], the angular-dependent probability of trans-cis isomerization per unit time, $p_{T}\left(\theta \right)$, can be written in the form proportional to $\cos^{2}\theta$:

(1) $$p_{T}\left(\theta \right)=P_{T}\cos^{2}\theta .$$

Here the constant $P_{T}$ is proportional to the intensity of the polarized light, *I*: $P_{T}=k_{TC}I$. The rate constant $k_{TC}$ is related to the absorption cross-section of the trans-isomer; the value $k_{TC}$ can be extracted from experimental data [99].

The cis-trans photoisomerization is well approximated as an angular-independent process due to isotropic polarizability tensor for a bent cis-isomer. Thus, one can write for the probability of cis-trans isomerization per unit time, $p_{C}\left(\theta \right)$:

(2) $$p_{C}\left(\theta \right)=P_{C},$$

where the constant $P_{C}$ is defined by two contributions [99]: $P_{C}=k_{CT}I+\gamma$. The first term is due to the photoisomerization process and the second one is defined by thermal transformation of excited cis-isomer to the ground trans-state.

Equations (1) and (2) define the probabilities of trans-cis and cis-trans photoisomerization processes. These equations can be used directly both in computer simulations and in theoretical studies. Below in Section 2.3 we will show, how the mentioned equations can be used in Monte Carlo simulations. In the present subsection of the review we discuss the theoretical formalism developed in Ref. [117] to describe the kinetics of photoisomerization and photo-induced orientation ordering.

In the framework of the theoretical formalism, the time evolution of the angular distribution functions, $n_{T}\left(\theta \right)$ and $n_{C}\left(\theta \right)$, for trans- and cis-isomers is defined by following kinetic equations [117]:

(3) $$\frac{\partial n_{T}\left(\theta \right)}{\partial t}=-p_{T}\left(\theta \right)n_{T}\left(\theta \right)+\int d\Omega^{\prime }p_{C}n_{C}\left(\theta^{\prime }\right)f_{CT}\left(\Omega^{\prime }\to \Omega \right)+D_{T}\nabla_{\theta }^{2}n_{T}\left(\theta \right)$$

(4) $$\frac{\partial n_{C}\left(\theta \right)}{\partial t}=\int d\Omega^{\prime }p_{T}\left(\theta^{\prime }\right)n_{T}\left(\theta^{\prime }\right)f_{TC}\left(\Omega^{\prime }\to \Omega \right)-p_{C}n_{C}\left(\theta \right)+D_{C}\nabla_{\theta }^{2}n_{C}\left(\theta \right).$$

The first and second terms in the right-hand sides of the last equations describe the contribution from the trans-cis and cis-trans isomerization processes, respectively. The variable Ω contains two Euler angles: Ω≡(θ,φ), where φ is the azimuthal angle which determines the orientation of the azobenzene around the polarization vector E. Integrations over the angles in Equations (3) and (4) are defined via the volume element as follows: dΩ=sinθdθdφ, θ∈[0,π] and φ∈[0,2π]. The functions $f_{TC,CT}\left(\Omega^{\prime }\to \Omega \right)$ determine the probability of stochastic reorientation of the chromophore during the trans-cis and cis-trans photoisomerization processes from the initial orientation $\Omega^{\prime }$ to the final orientation Ω. We assume that the azimuthal reorientation of the chromophore with respect to its initial orientation is random and $f_{TC,CT}$ are solely functions of the angle χ between the initial and final orientations of the chromophore: $f_{TC,CT}=f_{TC,CT}\left(\chi \right)$.

The last terms in Equations (3) and (4) describe the orientation diffusion of the trans- and cis-isomers; $D_{T}$ and $D_{C}$ are the rotational diffusion coefficients for trans- and cis-isomers, respectively. In Ref. [127], it was shown that the values $D_{T}$ and $D_{C}$ are very close to each other; therefore, below an approximation $D_{T}\approx D_{C}\equiv D$ will be used. Thus, the photoisomerization kinetics is described by a system of two integro-differential Equations (3) and (4), which are non-linear with respect to the angular variable. To solve these equations is not a trivial problem.

Alternatively, the photo-induced orientation ordering can be described in a more simple way by introducing an effective orientation potential acting on azobenzene chromophores. For equivalent description of the phenomenon the orientation potential should reproduce the orientation ordering which follows from the kinetic Equations (3) and (4). Note that both trans- and cis- isomers influence the conformations of azo-containing polymer chains and define the total mechanical stress and photo-induced mass transport. Therefore, the most important characteristics to be investigated is the orientation ordering of all isomers.

The angular distribution function of all chromophores, $n\left(\theta \right)=n_{T}\left(\theta \right)+n_{C}\left(\theta \right)$, affected by effective orientation potential $U_{\mathrm{eff}}\left(\theta \right)$, is determined by the following kinetic equation [117]:

(5) $$\frac{\partial n}{\partial t}=\frac{D}{\sin\theta }\frac{\partial }{\partial \theta }\left[\sin\theta \left(\frac{\partial n}{\partial \theta }+\frac{n}{kT}\frac{\partial U_{\mathrm{eff}}}{\partial \theta }\right)\right].$$

It is worth to calculate the orientation potential which appears at the moment when the light is switched on. The evolution of n(θ) at the initial stage can be described by the Taylor expansion:

(6) $$n\left(\theta ,t\right)=n\left(\theta ,t=0\right)+{\left(\partial n/\partial t\right)}_{t=0}\cdot t+\ldots$$

At the initial state in the dark the orientation distribution of chromophores is isotropic: n(θ,t=0)=1/(4π). The first derivative ${\left(\partial n/\partial t\right)}_{t=0}$ can be found from Equation (5) as follows:

(7) $${\left(\partial n/\partial t\right)}_{t=0}=\frac{D}{4\pi kT\sin\theta }\frac{\partial }{\partial \theta }\sin\theta \frac{\partial U_{\mathrm{eff}}}{\partial \theta }.$$

On the other hand, the first derivative ${\left(\partial n/\partial t\right)}_{t=0}={\left(\partial n_{T}/\partial t\right)}_{t=0}+{\left(\partial n_{C}/\partial t\right)}_{t=0}$ can be determined from photoisomerization kinetics described by Equations (3) and (4), that give:

(8) $${\left(\partial n/\partial t\right)}_{t=0}=\frac{P_{T}{\langle \sin^{2}\chi \rangle }_{TC}}{8\pi }\left(1-3\cos^{2}\theta \right).$$

For equivalency of the two approaches (based either on the angular-dependent photoisomerization or on the orientation potential) it is necessary to equate the right-hand sides of the Equations (7) and (8). It provides equivalent time evolution of the distribution function n(θ) in the framework of both approaches. Equating the right-hand sides of the Equations (7) and (8) we obtain the following differential equation for the potential $U_{\mathrm{eff}}\left(\theta \right)$ [117]:

(9) $$\frac{\partial }{\partial \theta }\sin\theta \frac{\partial U_{\mathrm{eff}}}{\partial \theta }=\frac{P_{T}kT{\langle \sin^{2}\chi \rangle }_{TC}}{2D}\left(1-3\cos^{2}\theta \right)\sin\theta .$$

After double integration of both sides of the last equation with respect to θ we obtain the solution for $U_{\mathrm{eff}}\left(\theta \right)$ in the following form [117]:

(10) $$U_{\mathrm{eff}}\left(\theta \right)=V_{0}\cdot \cos^{2}\theta ,$$

where the strength of the potential $V_{0}$ is related to the physical parameters controlling the photoisomerization process [117]:

(11) $$V_{0}=\frac{P_{T}kT{\langle \sin^{2}\chi \rangle }_{TC}}{4D}.$$

Note that the effective orientation potential in the form of Equation (10) was phenomenologically proposed by two independent groups [128,129] and was used later to describe theoretically the photoorientation in broad classes of azo-polymers [114,118,130,131,132,133,134,135,136,137,138]. Thus, the presented theoretical formalism based on the photoisomerization kinetics described by Equations (3)–(11) justifies the introduction of the orientation potential in the form of Equation (10). Furthermore, the presented formalism provides the value of the strength of the potential as a function of the physical parameters controlling the photoisomerization kinetics. In particular, one can see that the strength of the potential $V_{0}$ is proportional to the intensity of the light:

(12) $$V_{0}=C_{1}\cdot I,\;\mathrm{where}\;C_{1}=\frac{k_{TC}kT{\langle \sin^{2}\chi \rangle }_{TC}}{4D}.$$

Thus, we presented here two equivalent approaches to describe the photoorientation in azo-polymers under illumination with linearly polarized light. The first approach is based on the explicit consideration of angular-dependent photoisomerization process using kinetic Equations (3) and (4), which contain the absorption probabilities given by Equations (1) and (2). The second approach is based on the orientation potential described by Equation (10). In the next subsection of our review we present a possible generalization of these two approaches to study the photoorientation in azo-polymers illuminated with the elliptically polarized light.

#### 2.1.2. *Elliptically Polarized Light*

The elliptically polarized light represents an electromagnetic radiation, in which the tip of the electric field vector describes an ellipse within the plane perpendicular to the direction of the light propagation. The light-induced deformation under elliptically polarized light is of a special interest as it naturally appears in surface relief gratings which are inscribed by two orthogonally polarized light beams [93,139]. Theoretical formalism to describe the orientation ordering in azo-polymers illuminated with the elliptically polarized light can be developed as a generalization of the formalism presented in previous section for the linearly polarized light. It is well known that elliptically polarized light can be presented as a superposition of two linearly polarized waves propagating along the same direction (e.g., along the *z*-axis) with the perpendicular electric vectors:

(13) $$E_{x}\left(t\right)=E_{x,0}\cos\left(\omega t\right)\;\mathrm{and}\;E_{y}\left(t\right)=E_{y,0}\sin\left(\omega t\right).$$

The orthogonal *x*- and *y*-axes are directed along the principal axes of the ellipse described by the tip of electric field vector; the magnitudes of the two linearly polarized waves $E_{x,0}$ and $E_{y,0}$ are equal to the main semi-axes of this ellipse; ω is the angular frequency of the light. The intensities of the waves, $I_{x}$ and $I_{y}$, are related to the total intensity of the elliptically polarized light, *I*, as follows:

(14) $$I=\frac{c}{4\pi }\langle E^{2}\rangle =\frac{c}{4\pi }\langle E_{x}^{2}+E_{y}^{2}\rangle =I_{x}+I_{y},$$

where *c* is the speed of light in vacuum.

Since the trans-cis photoisomerization events generated by the two linearly polarized waves are independent processes, one can simply write the probability of the trans-cis isomerization $p_{T}$ under action of the elliptically polarized light as a sum of two contributions, using Equation (1):

(15) $$p_{T,\;\mathrm{elp}}=k_{TC}I_{x}\cos^{2}\theta_{x}+k_{TC}I_{y}\cos^{2}\theta_{y},$$

where $\theta_{x}$ and $\theta_{y}$ are the angles formed by the long axis of a chromophore with respect to the *x*- and *y*-axes, respectively. Using Equation (14), the probability of absorption can be rewritten as follows:

(16) $$p_{T,\;\mathrm{elp}}\left(\theta_{x},\theta_{y}\right)=P_{T}\cdot \left(w_{x}\cos^{2}\theta_{x}+w_{y}\cos^{2}\theta_{y}\right),$$

where $P_{T}=k_{TC}I$; the factors $w_{x}=I_{x}/I$ and $w_{y}=I_{y}/I$ are the relative contributions of the linearly polarized waves to the total light intensity ($w_{x}+w_{y}=1$) and are related to the aspect ratio of the ellipse described by the tip of the electric field vector.

Similarly, the effective orientation potential can be generalized for the elliptically polarized light. Each linearly polarized wave described by Equation (13) produces an effective orientation potential according to Equations (10)–(12). Thus, the effective potential of a chromophore under the elliptically polarized light can be presented as a superposition of the potentials produced by these two waves:

(17) $$U_{\mathrm{eff},\;\mathrm{elp}}\left(\theta_{x},\theta_{y}\right)=V_{0}\cdot \left(w_{x}\cos^{2}\theta_{x}+w_{y}\cos^{2}\theta_{y}\right),$$

where $V_{0}=C_{1}\cdot I$. It is important to note that according to Equations (16) and (17), the elliptically polarized light produces a biaxial ordering in general case, $w_{x}\neq w_{y}>0$.

The derived Equations (16) and (17) for the elliptically polarized light reproduce important limiting cases. For the linearly polarized light ($w_{x}=1$ and $w_{y}=0$), Equations (16) and (17) are reduced to corresponding relationships (1) and (10) derived in the previous subsection. For the circularly polarized light ($w_{x}=w_{y}=1/2$), Equations (16) and (17) are transformed as follows:

(18) $$p_{T,\;\mathrm{circ}}\left(\theta_{z}\right)=\frac{1}{2}P_{T}\sin^{2}\theta_{z}\;\;\mathrm{and}\;\;U_{\mathrm{eff},\;\mathrm{circ}}\left(\theta_{z}\right)=\frac{1}{2}V_{0}\cdot \sin^{2}\theta_{z},$$

where $\theta_{z}$ is the angle formed by the long axis of the chromophore with respect to the *z*-axis, which lies along the propagation of the light, $\cos^{2}\theta_{x}+\cos^{2}\theta_{y}+\cos^{2}\theta_{z}=1$. One can see from Equation (18) that circularly polarized light produces a uniaxial alignment of chromophores along the direction of light propagation. Relationship (18) is in agreement with the results of previous studies devoted specially to the circularly polarized light [119,126,140].

Thus, in the present subsection of our review we developed a theoretical formalism, which can be used in further investigations to study the photoorientation in azobenzene-containing polymers of various structure under illumination of elliptically polarized light by means of both theoretical methods and computer simulations.

#### 2.1.3. *Photo-Induced Mechanical Stress*

One of the most important application of the theoretical formalism based on the orientation potential introduced above is its ability to estimate the photo-induced mechanical stress. According to the monograph [4], the action of the orientation potential $U_{\mathrm{eff}}$ on the rod-like moieties leads to the appearance of the mechanical stress. Using Equation (9.52) of Ref. [4], the components of the stress tensor, $\sigma_{\alpha \beta }$, appearing in azo-polymer due to the reorientation of rod-like azo-moieties under action of the potential $U_{\mathrm{eff}}$ can be calculated as follows:

(19) $$\sigma_{\alpha \beta }=-n_{0}\langle {\left(u\times RU_{\mathrm{eff}}\right)}_{\alpha }u_{\beta }\rangle .$$

Here $n_{0}$ is the number density of the chromophores, u is the unit vector directed along the rod-like chromophore in the trans-state, R=u×(∂/∂u) is the rotational operator and the averaging is over orientation of all chromophores. Substituting the orientation potential given by Equations (10) or (18) into the Equation (19), one can see that the characteristic values of the mechanical stress are proportional to the factor $n_{0}V_{0}$. The numerical prefactors are determined by the averaged moments of the 4th order, $\langle u_{\alpha }^{2}u_{\beta }^{2}\rangle$, for the orientation distribution function of chromophores. For instance, as was shown in Ref. [117], the magnitude of the normal stress, $\sigma =\sigma_{xx}-\sigma_{yy}$, under action of the linearly polarized light (the light polarization E is directed along the *x*-axis) is given by $\sigma =0.4\;n_{0}V_{0}$.

Now, using Equation (11), which relates $V_{0}$ with the physical parameters controlling the photoisomerization in azo-materials, one can estimate the characteristic magnitude of the photo-induced mechanical stress. Parameter kT/D in Equation (11) is related to the viscosity of the material η [4]: $kT/D=\pi \eta L^{3}/\left[3\left(\ln2p-0.5\right)\right]$, where *L* and *p* are the length and the aspect ratio of the orienting moieties. Using typical values $\eta \approx 10^{3}\;\mathrm{GPa}\cdot s$ for materials near the glass-transition temperature $T_{g}$[141] and geometrical characteristics of the azobenzene chromophores L≈0.9 nm and p≈3, as well as the typical values $k_{TC}\approx 0.4\;{\mathrm{cm}}^{2}/J$ and $n_{0}\approx 1.5\times 10^{21}\;{\mathrm{cm}}^{-3}$ for azo-materials [99], one can estimate the characteristic magnitude of the normal stress at the light intensity $I=0.1\;{W/\mathrm{cm}}^{2}$ as follows [117]: $\sigma =0.4\;n_{0}V_{0}\leq 4$ GPa for materials near $T_{g}$. Here the maximal value of σ is given for the maximal value of the parameter $\langle \sin^{2}\chi \rangle =1$ in Equation (11), which is related to the angular jump χ of a chromophore during the trans-cis photoisomerization event.

Remarkably, the mechanical stress estimated above has the same order of magnitude as was estimated in experimental works [111,112,113] which showed that light-induced mechanical stress can reach a giant value of 2 GPa and is able to deform covalent bonds as well as to break metallic layers on the surface of a glassy azo-polymer. Thus, the presented theory explains unambiguously the possibility for the appearance of the photo-induced stress of such a giant magnitude. From the other side, the photo-induced stress due to the factor $\langle \sin^{2}\chi \rangle <1$ can be lower than 4 GPa. The angular jumps of the chromophores during the photoisomerization process are hindered in a glassy material by the stiffness of the surrounding media. At small redistribution angles $10^{\circ }\leq \chi \leq 20^{\circ }$, which can be estimated for the chromophores in glassy materials [117], we obtain the values for mechanical stress between 100 MPa and 500 MPa at $I=0.1\;{W/\mathrm{cm}}^{2}$. Note that the last values are above the typical values of the yield stress, $\sigma_{Y}$, for glassy polymers (e.g., $\sigma_{Y}\approx 50\;\mathrm{MPa}$ for PMMA). At $\sigma >\sigma_{Y}$ the glassy polymer deforms irreversibly. Thus, the proposed theory provides the physical background of the light-induced mechanical stress large enough to deform irreversibly glassy azo-polymers, the fact which is widely observed, e.g., in SRG experiments.

Recent theoretical work [119] presents a detailed description, how to investigate the time-dependent orientation state of polymer backbones and to calculate the light-induced stress under homogeneous irradiation. Implementing the stress into a viscoplastic material model of the finite element software ANSYS, the directional photodeformations of azo-polymer posts and micropillars under linearly and circularly polarized light could be reproduced in accordance with the experiments [103,104,142]. The viscoplastic modeling approach has been further developed [120] to describe the appearance of stripe patterns in a quickly moving azo-polymer film irradiated with a strongly focused laser beam [105,143].

To conclude the present section we note that according to Equations (17) and (19), the photo-induced mechanical stress under the elliptically polarized light is also defined by the factor $n_{0}V_{0}$ as for the linearly polarized light. Thus, the present work generalizes the results of the previous theory [117] devoted to linearly polarized light and shows that the magnitude of the mechanical stress under the elliptically polarized light can also achieve the values 100 MPa–4 GPa as for the linearly polarized light. The photo-induced mechanical stress of such giant magnitudes can induce molecular motion in azo-polymers which can be deep in the glassy state. As it will be discussed below, the last consequence of the theory can serve as a background for the Monte Carlo simulations which assume *a priori* the possibility of molecular motions even in glassy polymers under illumination with the light.

### 2.2. Degenerate Two Wave Mixing Experiment

**Figure 1:**
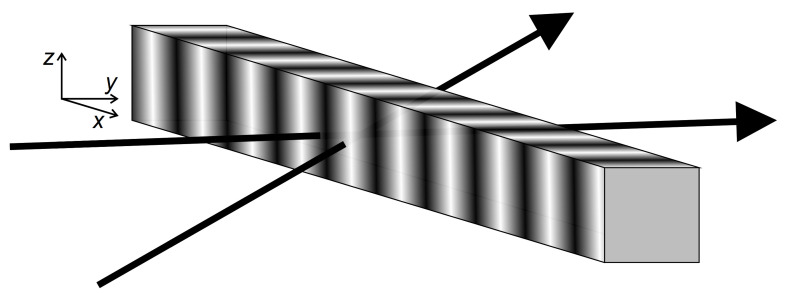
Scheme of the two-wave mixing experiment used for grating recording.

In the degenerate two-wave mixing (DTWM) experiment two coherent laser beams of arbitrary polarization interfere. The polarization of the emerging optical field varies spatially in magnitude and/or orientation. *s*-polarized beams at symmetric geometry, with Bragg’s planes perpendicular to the film surface, generate intensity fringe patterns (Figure 1) of the form:

(20) $$I=2\;I_{0}\left(1+\sin\left(qx\right)\right),$$

where $I_{0}$ denotes the intensity of each of the incoming beams, q=2π/Λ denotes the wave-vector of the grating, and Λ—its period.

The light diffraction efficiency $\eta_{s}$ in the case of two *s*-polarized beams is defined as

(21) $$\eta_{s}=\frac{I_{1}}{I_{0}},$$

where $I_{1}$ denotes the intensity of first-order diffracted light beam. In a small signal approximation, without absorption grating contribution to the diffraction efficiency, assuming that the anisotropic optical properties are related only to the molecules in *trans* state, $\eta_{s}$ becomes [144,145]

(22) $$\eta_{s}\propto {\left(\Delta n\right)}^{2},$$

where Δn denotes the amplitude of refractive index of a periodic sinusoidal grating. Similar equations hold for *p*-polarized light.

### 2.3. Monte Carlo Simulations of Host-Guest Systems in BFM Approach

#### 2.3.1. Metropolis Monte Carlo Method

Monte Carlo method uses random numbers to estimate parameters of a probe sampled from a general population. Metropolis Monte Carlo approach [146] was successfully used in polymer science [10,147]; other implementations in polymer science are also used [12,148,149].

Metropolis Monte Carlo algorithm samples a set of typical equilibrium configurations of a system of interacting physical objects at absolute temperature *T*. For this purpose, one starts from some (*old*) configuration with energy $E_{old}$ and modifies it by performing trial movements of its constituent elements. Trial configuration generated in this way has energy $E_{trial}$, and it is accepted as a new member of the set of configurations with probability equal to the smaller value of two expressions: 1 and $e^{-\left(E_{trial}-E_{old}\right)/\left(k_{B}T\right)}$, where $k_{B}$ denotes Boltzmann constant (Metropolis acceptance rule). If the trial configuration is rejected by this rule, then *old* configuration becomes the new member of the set of configurations. This procedure is iterated, starting from the last configuration added to the set. Chosen thermodynamic parameters are monitored to localize the onset of thermodynamic equilibrium. The configurations generated in the same way in equilibrium regime are used to calculate thermodynamic and structural parameters, as well as kinetic/dynamic effects.

#### 2.3.2. BFM Model in 2D and 3D

**Figure 2:**
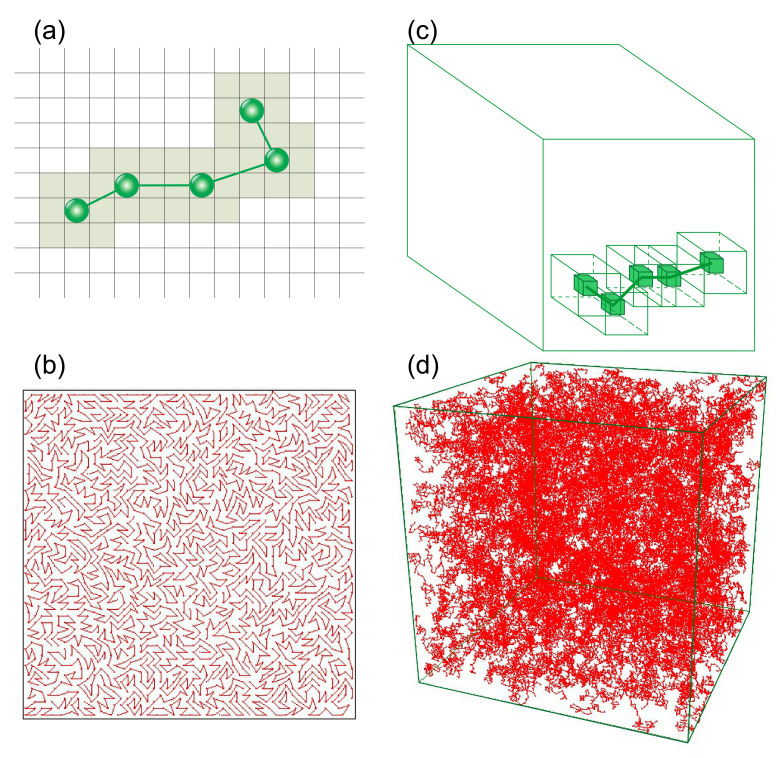
Lattice bond fluctuation model in 2D (**a**) and 3D (**c**). Exemplary configurations in 2D (**b**) and 3D standard system (**d**). Based on [150].

In 2D and 3D BFM models the neighboring monomers of a polymer chain are connected by bonds. This bonds are related to the Kuhn segments formed from groups of monomers along the polymer chain: averaged bond length in the BFM equals the length of the Kuhn segment [10]. The BFM model in 3D uses five bond orientations. Bond lengths read (in lattice constants) [20]: 2, $\sqrt{5}$, $\sqrt{6}$, 3, $\sqrt{10}$. The corresponding bond stretching energies $E_{i}\;\left(i=1,\ldots ,5\right)$ read $E_{i}=E_{0}\;\varepsilon_{i}$, where parameter $E_{0}$ sets an energy scale and defines the reduced temperature $T^{*}=k_{B}T/E_{0}$. In what follows we write, for simplicity, *T* instead of $T^{*}$. $\varepsilon_{i}$ represents a dimensionless energy equal $\varepsilon_{i}=\varepsilon =1$ for the first three bond lengths, and $\varepsilon_{i}$ = 0 for the remaining lengths. The glass transition temperature reads approximately $T_{g}=0.3$. In a single Monte Carlo step (MCS) each of the monomers performs the trial movement along one of randomly chosen directions x,y,z. It is accepted when the following three conditions are met: (i) a new length of the bond does not violate imposed restrictions, (ii) the trial position of the monomer is neither occupied by a monomer nor a nearest neighbour of a monomer and (iii) the Metropolis acceptance rule does not reject the movement. In most of our MC simulations in 3D, the polymer matrix is composed of $N_{0}=24,000$ polymer chains, each consisting of L=20 monomers, placed on $V_{p}=200\times 200\times 200$ lattice, which yields the reduced density $\rho =8N_{0}L/V_{p}=0.48$ (dense polymer melt). In what follows, we refer to it as a standard 3D matrix. In Figure 2 a scheme of the chain (Figure 2c) and an exemplary configuration of standard 3D matrix (Figure 2d) are presented.

For 2D model [40] the following set of bond lengths was used (in lattice constants): 2, $\sqrt{5}$, $\sqrt{8}$, 3, $\sqrt{10}$, $\sqrt{13}$; the corresponding bond stretching dimensionless energies $\varepsilon_{i}$ are 1, 0.64, 0.08, 0.02, 0.00, 0.15. In a single MCS each of the monomers performs a trial movement of unit length along one of the two directions x,y. The movement was accepted using the same procedure as for 3D system. In Figure 2a scheme of a single 2D chain (Figure 2a) and an exemplary configuration of 2D system (Figure 2b) are presented. 

**Figure 3:**
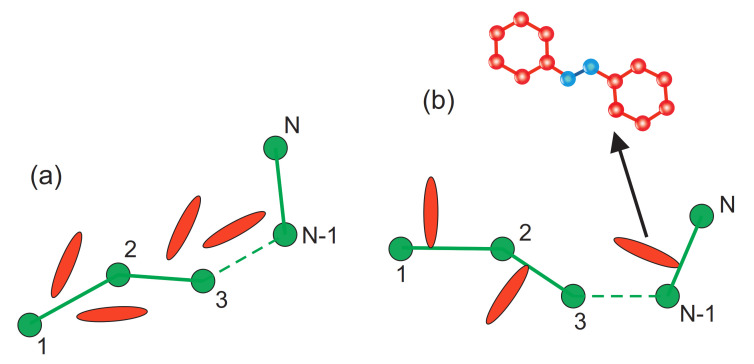
Azo-dyes embedded in the polymer matrix (**a**) and attached to BFM chain (**b**).

Typically, two kinds of host-guest systems are studied in experiments, with guest molecules (azodyes) embedded in the matrix or rigidly attached to the polymer chain. In the latter case, in BFM model, the azo-dye molecules are located close to one of the two monomers belonging to a bond. In trans state azo-dyes are perpendicular to the bond, see Figure 3.

#### 2.3.3. Local Characterization of the BFM Matrix

The dynamics of guest molecules depends, apart from the interaction with external fields, on its local static and dynamical neighbourhood. Two local parameters were introduced to characterize it.

**Figure 4:**
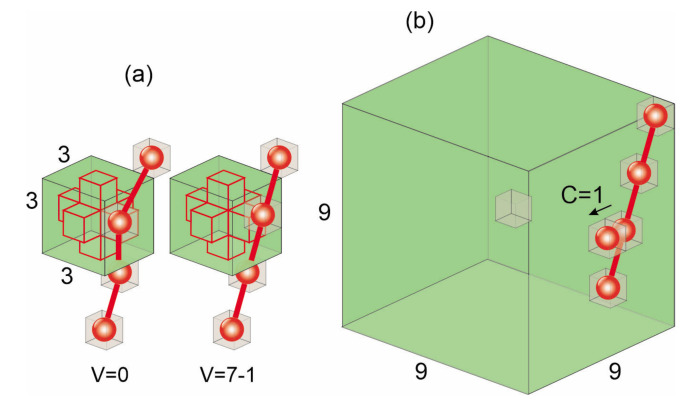
Scheme for calculation of local parameters *V* (**a**) and *C* (**b**)—see text for more details. Based on [157].

The first one, local void parameter $V(\vec{r})$ is a measure of local free volume around lattice site $\vec{r}$. This scalar field characterizes the inhomogeneity of the distribution of monomers in the system. It was introduced in Ref. [145] and extensively used to study host-guest systems [145,150,151,152,153,154,155,156]. To calculate the value of parameter $V(\vec{r})$ one analyzes the occupancy of a 3×3×3 cube around lattice site $\vec{r}$, see Figure 4a. The lattice site $\vec{r}$ corresponds to the central cell in the cube. The cross, which plays an important role in the model, consists of 7 cells: the central one and its 6 nearest neighbours. The lattice site $\vec{r}$ is blocked ($V(\vec{r})=0$) in the case when a monomer occupies one of the cells of the cross. In other cases each monomer in the cube (with the exception of the cross) decreases the value of parameter $V(\vec{r})$ by 1:

(23) $$V\left(\vec{r}\right)=V_{0}-k,$$

where $0\leq k\leq V_{0}$ stands for the number of monomers in the cube. Thus, *V* becomes a measure of local free volume. High values of $V(\vec{r})$ correspond to a neighbourhood with few (if any) monomers, while its low values indicate that lattice site $\vec{r}$ has some number of neighbouring monomers. We use $V_{0}=7$ because in the simulations the maximal occupancy of the cube by the monomers was equal 7. The linear counting rule, Equation (23) is rather arbitrary, but its modifications lead to rather quantitative and not qualitative changes of the results [145].

The correspondence between the size of the cube and the corresponding scale in real polymeric materials can be roughly estimated using the length of the Kuhn segment which corresponds to the averaged bond length in BFM. For example, the Kuhn’s length for PMMA is 1.7 nm [158] leading to the conclusion that the volume of the cube used to calculate *V* is approximately $10\;{\mathrm{nm}}^{3}$ [157].

The second parameter, $C(\vec{r})$, characterizes dynamical changes of the distribution of monomers in a close neighbourhood of point $\vec{r}$. To this end one monitors the total number $\delta n_{i}$ of changes of the occupations of lattice sites ${\vec{r}}_{i}$ close to $\vec{r}$, in a unit of MC time, see Figure 4b. In the case when the occupation of site ${\vec{r}}_{i}$ has changed, $\delta n_{i}=1$; otherwise $\delta n_{i}=0$. The formula for *C* reads [145,152]

(24) $$C=\sum_{i}\delta n_{i}.$$

The sum is taken over cells in a cube 9×9×9 around the lattice site $\vec{r}$.

#### 2.3.4. Light-Matter Interaction for Host-Guest Systems in BFM Approach Polarization Effects

**Figure 5:**
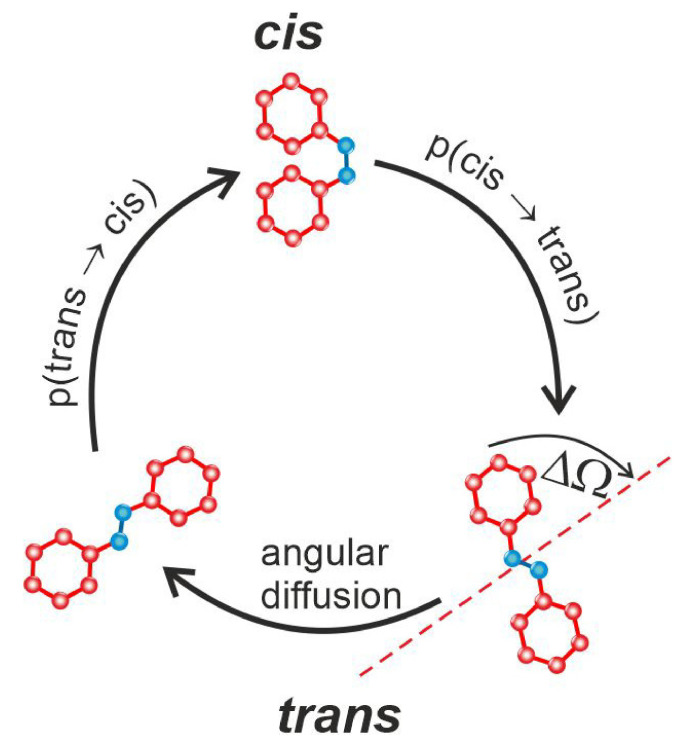
Scheme of *trans-cis-trans* cycle and angular diffusion process. Based on [150].

The kinetic MC modeling applies analytical expressions for transition probabilities (transition rates) *p* in MC unit time for kinetic processes. The kinetic model of host-guest systems includes the interaction of azo-dyes with an optical field (light-matter interaction), with the matrix and the intermolecular dye-dye interactions. The light-driven *trans-cis-trans* cycle of azo-dye molecules consists of three events: transitions trans→cis, cis→trans and angular diffusion (see Figure 5).

The concept of MC modeling was formulated in Ref. [144] for the case of pure light-matter interaction. The generalized transition rates for host-guest systems, which account for the matrix in the low concentration limit (when the dye-dye intermolecular interactions and the influence of the isomerization dynamics on the matrix can be neglected), were formulated in Refs. [145,152]. Namely, the transition rates for trans→cis, Equation (1), and cis→trans, Equation (2) transitions, were modified to account for the local matrix environment:

(25) $$p_{T}\left(\theta \right)=V\;I\;k_{TC}\cos^{2}\theta ,$$

(26) $$p_{C}\left(\theta \right)=V\;I\;k_{CT}.$$

The parameter

(27) $$R=\frac{k_{TC}}{k_{CT}}$$

plays an important role for the kinetics of host-guest systems [155,159,160].

In the case of two photon absorption the transition rate reads [161]:

(28) $$p_{T}\left(\theta \right)=VI^{2}k_{TC}^{\left(2\right)}\cos^{4}\theta .$$

In the case of all-optical poling transition rates include one- and two-photon absorptions, as well as the term which breaks the centrosymmetry: $p\left(\theta \right)\propto \cos^{3}\theta$ (see Section 3.7). The resulting transition rate is a weighted sum of those rates. For optimal conditions (equal excitation probabilities through one- and two-photon absorptions) [162] the rate of trans→cis transition reads, in the presence of the matrix [150]:

(29) $$p_{T}=V\;I\;p_{tc}\;\left(\cos^{2}\theta +\cos^{4}\theta +2\cos^{3}\theta \right).$$

The transition rate for rotational diffusion, from the orientation Ω=(θ,ϕ) to Ω+ΔΩ=(θ+Δθ,ϕ+Δϕ) (θ and ϕ denote polar and azimuthal angles, respectively), reads [145,152]:

(30) $$p\left(\Omega \to \Omega +\Delta \Omega \right)=\left\{\begin{array}{cc} p_{diff}\;V\;C & ,\;\Delta \Omega <\Delta \Omega_{0}\\ 0 & ,\;\Delta \Omega >\Delta \Omega_{0}, \end{array}\right.$$

where $p_{diff}$ is a constant. In the above formulas 0≤I≤1 denotes a dimensionless light intensity. In most of our MC simulations the intensity 0≤I(x)≤1 is a dimensionless quantity [163]. Its relation to “physical” intensity can, in principle, be made, given the relation between 1 MCS and the unit of time. To summarize, in the BFM the azo-dye molecules couple to the polymer matrix through the local parameters *V* and *C*.

Transition rate $p_{T}$ depends on the orientation of the dye and it can vary along the chain. Thus, the polarization of the light becomes an important factor for the dynamics of host-guest systems. The counterpart of the Full Polarization Model (FP model), Equation (25), is the Polarization Independent Model (PI model) which is insensitive to polarization—the transition probability $p_{T}$ is constant and independent on the orientation of an azo-dye. Both models were recently studied in 2D case [164]:

(31) $$p_{T}=\left\{\begin{array}{cc} I(x,y), & \mathrm{Polarization}\mathrm{Independent}(\mathrm{PI})\mathrm{Model},\\ I\left(x,y\right)\cos^{2}\theta , & \mathrm{Full}\mathrm{Polarization}(\mathrm{FP})\mathrm{Model}. \end{array}\right.$$

#### 2.3.5. Details of MC Simulations

In the host-guest BFM with azo-dyes attached to the polymer chain, the monomers perform two types of trial MC movements: thermal [40] and non-thermal, the latter related to the interaction of azo-dyes with light [154,163]. Thermal trial movements drive the matrix towards equilibrium and were described in Section 2.3.2.

Non-thermal MC movements model the action of forces and torques which have their origin in mechanical effect of photoisomerization cycles trans↔cis [154,163], and drive the system out of equilibrium. A photoisomerization transition of an azo-dye molecule grants an additional, non-thermal trial movement of the monomer closest to the dye. It has unit length and is done along one of randomly chosen directions x,y,z. It is accepted provided that the conditions (i) and (ii) are satisfied. Metropolis acceptance rule is no more valid since the trial movement is not thermally driven. This conclusion reflects the fact that the typical energy of light quanta which cause the photoisomerization transition (a few eV) much exceeds characteristic thermal energy at room temperature *T* ($k_{B}T\approx 3\times 10^{-2}$ eV).

The kinetics of cis→trans transitions is not taken into account directly in the model—after the photoisomerization transitions the molecules return to their trans states.

For a system consisting of $N_{0}$ monomers, a sweep consisting of $N_{0}$ trial movements, including both thermal and non-thermal ones, corresponds to one MCS, which represents a unit of MC “time” *t*. To avoid correlations, the monomers were selected randomly.

### 2.4. Characterization of Physical Effects: Parameters

#### 2.4.1. Diffraction Efficiency

Diffraction efficiency $\eta_{s}$ is proportional to ${\left(\Delta n\right)}^{2}$, see Equation (22). Parameter Δn is proportional to Δ, which is given by the formula [144,145]

(32) $$\Delta =N^{trans}\left(x_{max}\right)\left(1+2\langle P_{2}\rangle \left(x_{max}\right)\right)-N^{trans}\left(x_{min}\right)\left(1+2\langle P_{2}\rangle \left(x_{min}\right)\right).$$

$N^{trans}$ denotes the concentration of *trans* molecules at *x* and $\langle P_{2}\rangle \left(x\right)$ is the local orientation parameter $P_{2}$ (second rank Legendre polynomial) averaged over y,z directions. Both parameters are calculated from Monte Carlo simulations. $x_{min}$ and $x_{max}$ denote local positions corresponding to minimal and maximal values of light intensity, respectively.

#### 2.4.2. SHG Efficiency

The quality of polar order in a system of quasi 1D charge transfer (CT) chromophores in thin films of polymer matrix determines the efficiency of the second harmonic generation (SHG). Experimentally, it is created using electric field corona poling, photo-assisted electric field poling or all optical poling [162] techniques. For such systems the second order non-linear optical (NLO) susceptibility is characterized by two tensor components (for details see e.g., Ref. [165]): the diagonal one

(33) $$\chi_{ZZZ}^{\left(2\right)}\left(-2\omega ;\omega ,\omega \right)=NF\beta_{zzz}\left(-2\omega ;\omega ,\omega \right)\langle \cos^{3}\theta \rangle ,$$

and the off diagonal one

(34) $$\chi_{XXZ}^{\left(2\right)}\left(-2\omega ;\omega ,\omega \right)=\frac{1}{2}NF\beta_{zzz}\left(-2\omega ;\omega ,\omega \right)\langle \sin^{2}\theta \;\cos\theta \rangle ,$$

where *Z* denotes the poling field direction (perpendicular to the thin film surface) and X is perpendicular to it. θ denotes an angle which the molecular axis *z* makes with *Z* direction, *N* denotes the number density of the chromophores (dipoles), $\beta_{zzz}$—molecular first hyperpolarizability of CT chromophores and *F*—the local field factor. The brackets 〈…〉 in Equations (33) and (34) denote an average over the orientations of all chromophores. MC dynamics of the build-up of non-linear susceptibility is monitored using load parameter

(35) $$L=N<\cos^{3}\theta >,$$

which is proportional to $\chi_{ZZZ}^{\left(2\right)}$. The average $<\cos^{3}\theta >$ plays the role of polar order parameter.

#### 2.4.3. Displacement Complex Dynamics

The dynamics of single polymer chains is characterized using the displacement of their center of mass (CM). To this end, the vector ${\vec{r}}_{1}^{\left(CM\right)}\left(t\right)$ of CM of a single chain is calculated after each MC step. Important parameter is the square of the displacement of CM from the initial position at t=0: ${\left(\Delta {\vec{r}}_{1}^{\left(CM\right)}\right)}^{2}\left(t\right)={\left({\vec{r}}_{1}^{\left(CM\right)}\left(t\right)-{\vec{r}}_{1}^{\left(CM\right)}\left(0\right)\right)}^{2}$. To improve the statistics of the data, the simulations were repeated for a statistical ensemble of $N_{0}=10^{3}$ independent chains, and single squared displacements ${\left(\Delta {\vec{r}}_{i}^{\left(CM\right)}\right)}^{2}\left(t\right),\;\left(i=1,\ldots ,N_{0}\right)$ were averaged over this ensemble to obtain the squared displacement ${\left(\Delta {\vec{r}}^{\left(CM\right)}\right)}^{2}\left(t\right)$ of CM:

(36) $${\left(\Delta {\vec{r}}^{\left(CM\right)}\right)}^{2}\left(t\right)=\frac{1}{N_{0}}\sum_{i=1}^{N_{0}}{\left(\Delta {\vec{r}}_{i}^{\left(CM\right)}\right)}^{2}\left(t\right).$$

This parameter characterizes the random walk of CM of a single chain. A similar procedure was used for the polymer matrix; the averaging was done over all the chains in the matrix.

The exponent γ in the power law

(37) $${\left(\Delta {\vec{r}}^{\left(CM\right)}\right)}^{2}\left(t\right)\propto t^{\gamma }$$

classifies three types of complex dynamics: subdiffusion (γ<1), normal diffusion (γ=1) and superdiffusion (γ>1).

## 3. Results

### 3.1. Polymer Chain Motion in Orientation Approach

The theoretical approaches, presented in Section 2.1, which use either angular-dependent kinetics of photoisomerization or the effective orientation potential, were successfully applied to describe the photo-deformation and ordering in azobenzene-containing polymers of various structures, including amorphous polymers [114,117,130,131], crosslinked isotropic polymer networks [132,133] and anisotropic azobenzene-containing liquid crystalline (LC) polymer networks [118,134,135,136,137,138]. The proposed theories can explain various experimental results for broad classes of azobenzene-containing polymers, as summarized briefly below.

As it was discussed in Section 2.1.3, the orientation potential provides the mechanical stress of the magnitude 100 MPa—4 GPa at conventional light intensity $I∼100\;{\mathrm{mW}/\mathrm{cm}}^{2}$, the stress being enough to deform irreversibly azobenzene polymers, which are deep in a glassy state [111,112,113,114,117,130,131]. Depending on the orientation distribution of azobenzene moieties inside polymer chains, preferably perpendicular or along the backbones of polymer chains, azo-polymers demonstrate either expansion or contraction along the polarization vector of the light [132,133,166,167]. Theoretically predicted dependence of the light-induced deformation of azobenzene-containing polymer networks on the degree of cross-linking as well as on the concentration of azobenzene chromophores [132,133] agrees well with experiments devoted to light-induced deformation [168]. Orientation interactions between the rod-like chromophores in trans-state and included LC mesogenic fragments result in additional orientation ordering in the plane perpendicular to the polarization vector of the light leading to the biaxial ordering and deformation of azobenzene containing LC polymers [134,135,169,170,171]. The last result is confirmed also by computer simulations [172].

### 3.2. Complex Structure of Local Free Volume in Polymer Matrix

The distribution in space of local free volume, characterized by local void parameter *V*, displays complex fractal-like structure [157] and leads to a quasi *binary* physical picture of the structure of an instantaneous configuration: about half of cells are blocked by monomers, while approximately one third of them have their neighbourhood nearly free of monomers. Cells with V=6 form scale-free clusters with fractal dimension dependent on the size of the cluster. Locally, they are characterized by exponentially decaying self correlation functions. In what follows we present the main results of Ref. [157].

#### 3.2.1. Mosaic-Like States: Maps of $V(\vec{r})$ and Correlations

**Figure 6:**
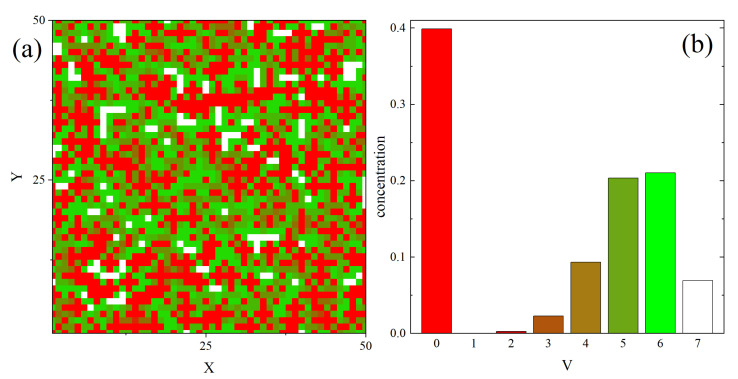
(**a**) Map $V(\vec{r})$ of *mosaic-like* free volume configurations of the matrix, see text for more details. (**b**) Probability distribution (empirical histogram) ρ(V) of local void parameter *V*. T=0.25. Based on [157].

Figure 6a shows a map $V(\vec{r})$ calculated on the basis of an instantaneous 2D section of 3D system. It displays a complex mosaic of red (V=0), green (V=5,6) and white cells (V=7) cells. They display a tendency for clustering; red clusters are usually bigger and separated from white ones.

The probability distribution ρ(V) of random variable *V*, represented by a normalized empirical histogram and shown in Figure 6b, displays a quasi two-peak structure. One peak corresponds to V=0 (low degree of local free volume), the other—to a group consisting of V=5,6,7 (high degree of local free volume). Blocked cells (V=0) constitute approximately 40% of the system; cells with high degree of local free volume (V=5,6,7)—correspondingly 21%,22% and 7%.

The distribution in space of cells with various values of *V* is not random. This fact was verified by calculating the probability for finding *k* cells with fixed value of *V* in a cube consisting of *n* cells, given the probability *p* that a single cell has free volume *V*, and comparing the results with Bernoulli distribution [173] $B\left(n,k,p\right)=\frac{n!}{(n-k)!k!}p^{k}{\left(1-p\right)}^{n-k}$, which corresponds to random (uniform) spatial distribution of cells. Theoretical and experimental (simulations) distributions were clearly distinct. This result implies an existence of spatial correlations in the distribution of cells.

The spatial correlations between cells with given value of local free volume *V* were studied using a generalization of pair correlation functions from the theory of liquids [174]. Namely, the correlation function $g_{2}\left(r,V_{1},V_{2}\right)$ is the conditional probability to find a cell with $V=V_{1}$ at distance *r* from a cell with $V=V_{2}$.

**Figure 7:**
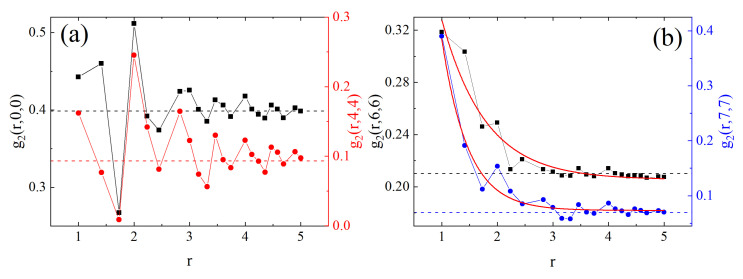
(**a**) Self-correlation functions $g_{2}\left(r,V,V\right)$ for V=0 (black squares, left *y* scale) and V=4 (red dots, right *y* scale). (**b**) Plot of $g_{2}\left(r,V,V\right)$ for V=6 (black squares, left *y* scale) and V=7 (blue dots, right *y* scale). Dashed lines: global concentrations of cells with corresponding values of *V*. Solid lines: exponential fits. T=0.25. Based on [157].

Of special interest are self-correlation functions $g_{2}\left(r,V,V\right)$. Two different types of self-correlations are present in the system. In the case of V=0 and V=4, the self-correlation functions display an oscillatory character, typical for simple dense liquids, see Figure 7a. The second type of self-correlations is typical for cells with a large amount of free volume (V=6 and V=7) and is characterized by an exponential decay

(38) $$g_{2}\left(r\right)=Ae^{-r/r_{0}}+const,$$

with superimposed oscillations (Figure 7b). Parameter $r_{0}$ reads 0.79 for V=6 and 0.45 for V=7; the correlation functions become constant at distances $r_{s}\approx$ 2.5–3. Those results can be interpreted in the following way: a sphere with diameter (in lattice units)

(39) $$D=2r_{s}\approx \left(5–6\right)$$

has few (if any) monomers inside it. The relation of parameter *D* to distances in a real system (polymethylacrylate) was roughly estimated in a similar way as in Section 2.3.3, leading to

(40) D=(6–9)nm

#### 3.2.2. Clusters: Configurations, Distribution, Fractal Features

**Figure 8:**
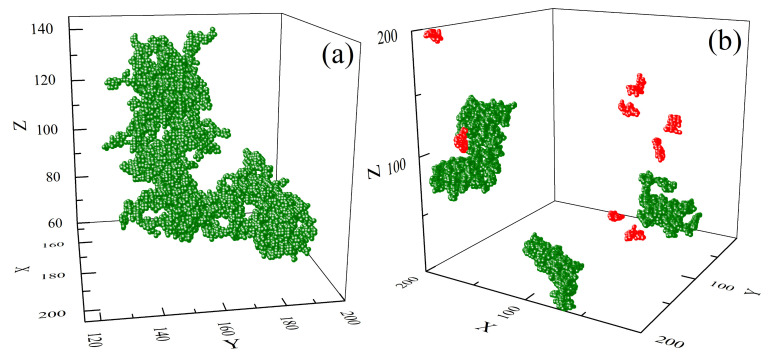
(**a**) Snapshot of a cluster of cells with V=6 (7614 cells). (**b**) Snapshots of clusters of cells with V=5 (red dots, approximately 100 cells) and of clusters with V=6 (green dots, size (3–7) × 10${}^{3}$ cells) in the simulation box. T=0.25. Based on [157].

Self-correlation functions indicate that some kind of clustering of cells with V=6 and V=7 is present in the system. By the definition, a cluster is a set of such cells that each of them is a nearest neighbour of at least one element of the set. Snapshots of configurations of chosen clusters are shown in Figure 8. The clusters of cells with V=6, in what follows referred to as 6-clusters, are very different from other types of clusters. First of all, they are much larger—the largest of them contain approximately of $10^{4}$ cells; the largest linear size of 6-clusters was approximately one half of the simulation box. The cluster shown in Figure 8a consists of 7614 cells and displays a complex spatial organization of the cells. Figure 8b shows a different large-scale (simulation box) organization of the largest 6-clusters (green, a few thousand cells) and 5-clusters (red, approximately 200 cells)—rather unexpected result, because the concentrations of cells with V=5 and V=6 are approximately equal (Figure 6). At the same time, the correlation functions for both types of local free volume are qualitatively different.

**Figure 9:**
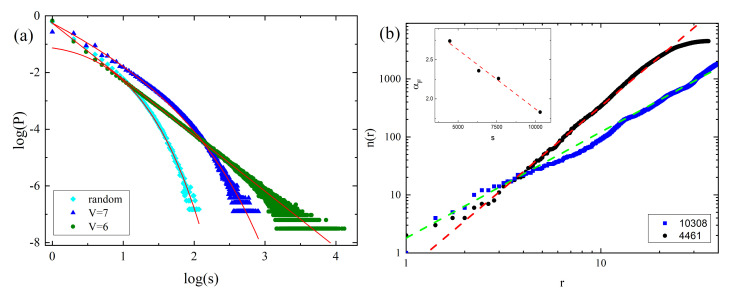
(**a**) Double logarithmic plot of probability distribution $p_{V}\left(s\right)$ of sizes *s* of *V*-clusters. *random*: Reference system of randomly distributed cells. Solid line: fits, Equation (41). (**b**) Double logarithmic plot of number of cells n(r) in a 6-cluster as a function of the distance *r*, see text for more details. Red and green dashed lines: power-law fits with $\alpha_{F}=2.74$ and 1.83, respectively. Inset: plot of fractal dimension $\alpha_{F}$ as a function of cluster size *s*. T=0.25. Based on [157].

The clusters vary in size. Figure 9a shows the double logarithmic plot of probability distribution $p_{6}\left(s\right)$ and $p_{7}\left(s\right)$ of number of cells (sizes) *s* in 6- and 7-clusters, respectively. For comparison, a distribution for a reference system which consists of randomly distributed cells with concentration 20%, is also plotted. Clearly, the sizes of 6- and 7-clusters are distributed in a different way than the sizes of random clusters. The empirical distributions $p_{V}\left(s\right)$ were fitted with the function well-known from percolation theory [175]:

(41) $$p\left(s\right)=s^{-\theta }\cdot a^{s}.$$

The distribution of 6-clusters (green circles) displays a surprising feature—the double logarithmic plot is linear, indicating a presence of a power law:

(42) $$p_{6}\left(s\right)\propto s^{-\theta },$$

with θ=2.14. To conclude, the clusters of nearly free cells (V=6) are scale-free—this property is characteristic for complex systems and is often accompanied by some kind of fractal organization. Figure 9b shows the double logarithmic plot of the number of cells n(r) which are at distance *r* from the center of a 6-cluster for two large clusters. The plots have a form of power law:

(43) $$n\left(r\right)∼r^{\alpha_{F}},$$

where $\alpha_{F}$ stands for the fractal dimension. Exponent $\alpha_{F}$ depends on the size of a 6-cluster—available simulation data suggest a linear dependence (see inset in Figure 9b):

(44) $$\alpha_{F}\left(s\right)=-1.52\times 10^{-4}s+3.38.$$

The largest clusters have fractal dimension lower than the fractal dimension $\alpha_{P}=2$, characteristic for clusters consisting of cells randomly distributed in three dimensions, below the percolation threshold [175]. For example $\alpha_{F}=1.83$ for 6-cluster with 10,308 cells. Equation (44) predicts that $\alpha_{F}=\alpha_{P}$ for $s\approx 9\times 10^{3}$. Smaller clusters have larger fractal dimension—a 6-cluster with 4461 cells is characterized by exponent $\alpha_{F}=2.74$, close to non-fractal limit $\alpha_{F}=3$. The latter, according to Equation (44), corresponds to 6-clusters with around 2500 cells

The results presented in this section remain valid in a wide temperature interval around glass transition temperature.

### 3.3. Inscription of Surface Relief Grating

Surface Relief Grating (SRG) [176,177] is a periodic surface corrugation pattern generated at temperatures well below $T_{g}$ in thin films of azobenzene functionalized polymers, using DTWM method [162]. Photoinduced mass transport has its origin in the dynamics of polymer chains, which results from light-promoted trans↔cis photoisomerization cycles of azobenzene dyes attached to the chains. The quantification of this hypothesis constitutes a challenge. An impressive number of theoretical and numerical approaches was used to study this topic, including mean-field approach [87], pressure gradient scenario [84,178], photoexpansion and photocontraction effects [179], viscoelastic flow [180], inchworm–like motion [86], gradient force models [85,181], Navier–Stokes dynamics [182,183], random–walk approaches [91], stochastic models [92,124,125], light-induced softening [85,95], atomistic molecular dynamics simulations [184], orientational approach [102,114] and others, see review papers [95,185] and Ref. [162].

A simple stochastic model of photoinduced mass transport was formulated in Ref. [154], using generalized BFM model. It treats both components—azo-dyes and the matrix—on the same footing, as parts of a host-guest system. The model demonstrated the photoinduced mass transport from bright to dark places of the illumination pattern and predicted experimentally confirmed presence of fine structure of SRG. The details are as follows.

The generalized MC BFM model was introduced in Section 2.3.5. The dependence on angle θ, Equation (25), was simplified by excluding from the photoisomerization transition only those azo-dyes which were nearly perpendicular to the vector of polarization of light. As a result, the model is weakly dependent on light polarization and corresponds rather to Polarization Independent Model than to Full Polarization Model, introduced in 2D case, see Equation (31). The length of a typical run was $10^{5}$ MCS.

**Figure 10:**
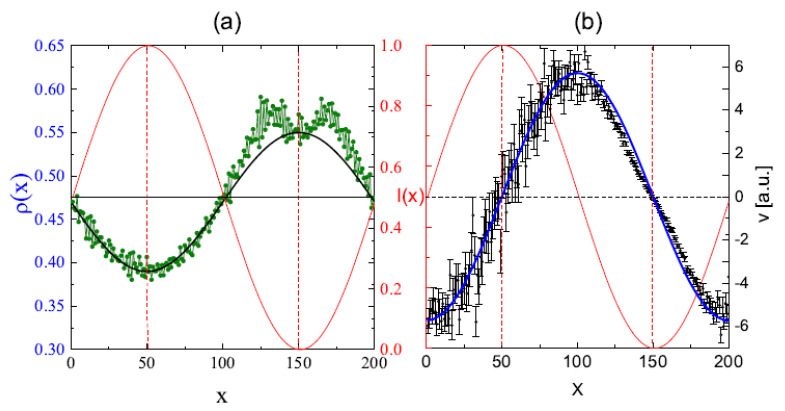
(**a**) Plot of monomer density profile ρ(x) at the end of the simulation and plot of scaled sine function (black solid curve). (**b**) Plot of macroscopic velocity $v_{x}$ of the CM of the chains, fitted with scaled gradient of the illumination ∇I (blue solid curve). Red line—the light illumination pattern I(x).

Two processes are responsible for the inscription of SRG. The primary process, due to the photo-driven chain mobility resulting from spatially modulated light intensity I(x), is responsible for the build-up of density grating, without surface corrugation. The corresponding monomer density profile ρ(x) at the end of the simulation is shown in Figure 10a. Initially, the system was homogeneous: ρ(x)=0.48. The effect of photoinduced mass transport is evident: the density decreases in strongly illuminated regions and increases in weakly illuminated ones. Close to the minimum of light illumination an overall sinusoidal density ρ(x) displays an additional two-peak structure. This effect is general, but its magnitude can depend on the parameters of the polymer as well as on the polarization setup [186]; in particular, it can be negligibly small. The directed movement of the chains is characterized by an averaged over chains component $v_{x}$ of the velocity vector $\vec{v}$ of centers of mass (CM) of the chains. Figure 10b shows the plot of $v_{x}\left(x\right)$ in the dominant period of the process of build-up of the density grating. It turns out that

(45) $$v_{x}\propto -\nabla I,$$

which reproduces a phenomenological relation postulated in macroscopic theories [84,85,178,181].

**Figure 11:**
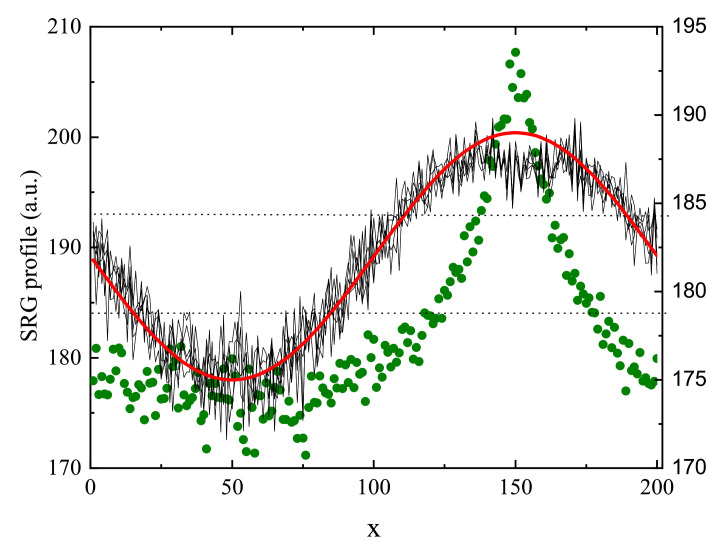
SRG profile in two limiting cases: weak stabilizing forces, end of the simulation (green circles, scale left) and strong stabilizing forces, the initial phase of build-up of SRG (thin black solid line, scale right). Thick red solid line: scaled sine function, dashed horizontal lines: initial surface profiles.

The secondary process creates the surface corrugation which depends on the mechanical stability of the surface. In MC model the stabilizing surface forces are modeled through non-thermal trial movements in the direction towards the bulk, applied to the monomers close to the surfaces [187]. The amplitude of the surface force determines the time delay between build-up of the density grating and of SRG grating, giving rise to various dynamical scenarios. In particular, when this force is weak, primary and secondary processes occur nearly simultaneously; as the result, the SRG profile is non-sinusoidal, without valleys at the top (Figure 11). This prediction is confirmed by the experimental results of SRG in azo-functionalized poly(esterimide) [188]. On the contrary, when the stabilizing forces are strong, both processes are separated in time and the build-up of the SRG takes place upon a well-developed density profile ρ(x), resulting in a nearly sinusoidal surface profile with a minor double-peak structure. In the model the two-peak structure is a transient effect, while in real experiments structure with double peak can be observed as a quasi-permanent or transient effect, depending on the magnitude of the time scales [188,189].

### 3.4. Complex Dynamics of Photoinduced Mass Transport in 3D and 2D

Although the model of the inscription of SRG discussed briefly in previous section demonstrated the directed photoinduced mass transport, it did not characterize typical motion of polymer chains. This missing information is essential for a deeper understanding of this process, as well as for its theoretical modeling. To gain some intuition concerning this topic, the MC dynamics of centers of mass of chains, represented by squared displacement ${\left(\Delta r\right)}^{2}$ (Section 2.4.3) was studied in 2D [164] and 3D [163] systems. It is well-known that the dynamics of a polymer chains varies with the time interval [4]. Since the process of the inscription of SRG requires approximately $10^{4}$ MCS, this MC interval was used in the simulations. 3D model uses the simplified version of transition probability $p_{T}$ discussed in previous section.

#### 3.4.1. 3D

**Figure 12:**
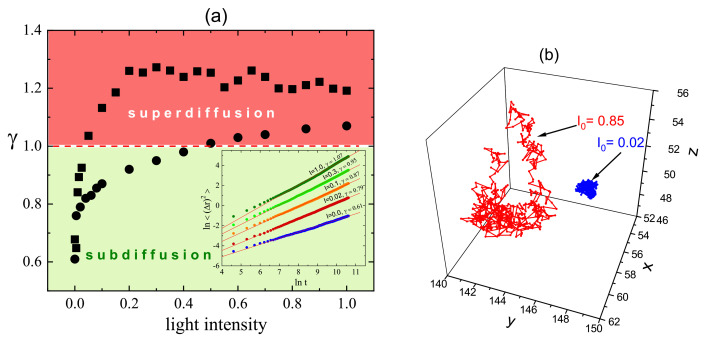
(**a**) Plot of exponent γ as function of the reduced light intensity $I_{0}$ for bulk system (circles) and for a single chain (squares). Inset: plots of $\ln\langle {\left(\Delta r\right)}^{2}\rangle$ against lnt for bulk system. (**b**) Exemplary trajectories of CM of chains for different illumination intensity, $I_{0}=0.85$ and $I_{0}=0.02$. Constant illumination, T=0.3. Based on [163].

Figure 12a shows the plot of exponent γ (Equation (37)) calculated from double-logarithmic plots of ${\left(\Delta r\right)}^{2}\left(t\right)$ (inset) for the polymer matrix used for SRG inscription. This time, however, the intensity of light was constant: $I\left(x\right)=I_{0}$. As expected [4], a purely polymeric system in the darkness displays a sub-diffusive behavior: γ≃0.61. As the intensity $I_{0}$ increases, a crossover from sub-diffusion (γ<1) through normal diffusion (γ=1) to super-diffusion (γ>1) is observed. The typical trajectories of CM, which correspond to sub-diffusive and super-diffusive dynamics, are shown in Figure 12b—the former is strongly localized, while the latter displays both localized and ballistic-like character.

Those results put forward a physical picture of the dynamics of SRG as corresponding to a dynamical coexistence of all three regimes of diffusion.

The dynamics of the chains in the bulk is determined by two factors: “self” dynamics of free chains and steric hindrances. Thus, it is imperative to analyze the motion of single chains. Plot of exponent γ as function of $I_{0}$ (Figure 12a) for a single chain (in an empty box) at constant illumination resembles qualitatively the plot for its bulk counterpart. However, the threshold intensity separating the sub-diffusive and super-diffusive dynamics, is lower. Exponent γ is larger for single chains than for the bulk system—it is the effect of steric hindrances.

**Figure 13:**
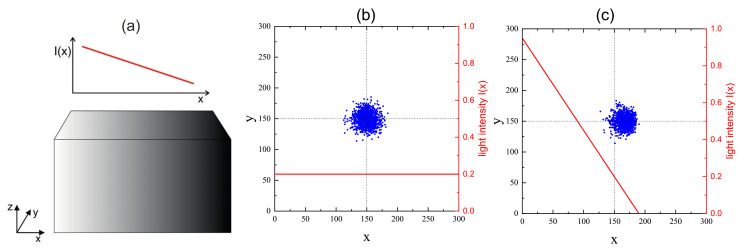
(**a**) Model of the system and illumination pattern. Projection of CM of $10^{3}$ chains onto *x*-*y* plane at the end of the simulation for $I_{0}=0.2$ and ∇I=0 (**b**) and ∇I=0.005 (**c**). T=0.25. [163].

The dynamics of chains at constant illumination is isotropic—the directed mass transport is absent. The essential features of the dynamics of an azo-polymer chain in inhomogeneous optical field were studied in the case of constant gradient of light intensity along the *x*-axis:

(46) $$I\left(x\right)=I_{0}-\nabla I\;\left(x-x_{0}\right),$$

where $x_{0}$ denotes center of lattice in the *x* direction and $I_{0}=I\left(x_{0}\right)$ is the intensity offset (Figure 13a). The linear inhomogeneity is the origin of the photo-driven mass transport, illustrated in Figure 13b,c which display the x−y projections of CM of $10^{3}$ independent polymer chains (initially at the center of the simulation cell) at the end of the simulation.

The dynamics in parallel direction *x* and in transverse directions y,z, is characterized, by analogy with Equation (37), in terms of exponents $\gamma_{x},\gamma_{y}$ and $\gamma_{z}$:

(47) $${\left(\Delta x\right)}^{2}\left(t\right)\propto t^{\gamma_{x}},\;{\left(\Delta y\right)}^{2}\left(t\right)\propto t^{\gamma_{y}},\;{\left(\Delta z\right)}^{2}\left(t\right)\propto t^{\gamma_{z}}.$$

**Figure 14:**
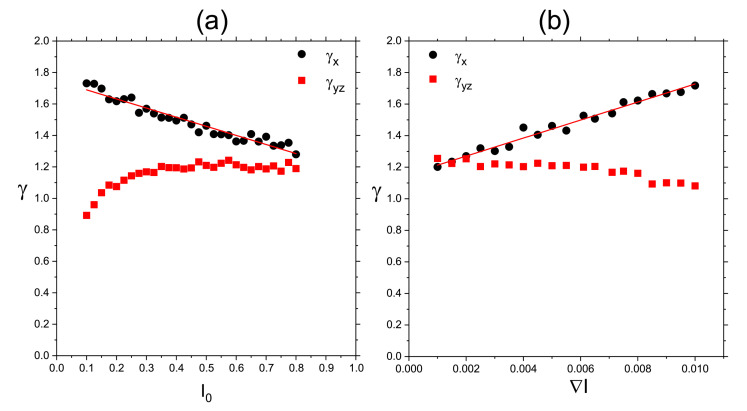
Plot of exponents $\gamma_{x}$ (black circles) and $\gamma_{y,z}$ (red squares) as functions of intensity offset $I_{0}$ for ∇I=0.005 (**a**) and of gradient of intensity ∇I for $I_{0}=0.5$ (**b**). T=0.25.

Figure 14 shows the plots of exponents $\gamma_{x}$ (black circles) and $\gamma_{y,z}$ (red squares) in function of intensity offset $I_{0}$ (a) and of the gradient of intensity ∇I. In both cases the parallel motion of CM is super-diffusive. Rather unexpectedly, exponent $\gamma_{x}$ depends linearly on the parameters—the origin of this effect remains unclear. The transverse exponents are approximately equal, $\gamma_{x}\neq \gamma_{y}\approx \gamma_{z}$, and correspond to super-diffusion for $I_{0}>0.1$.

**Figure 15:**
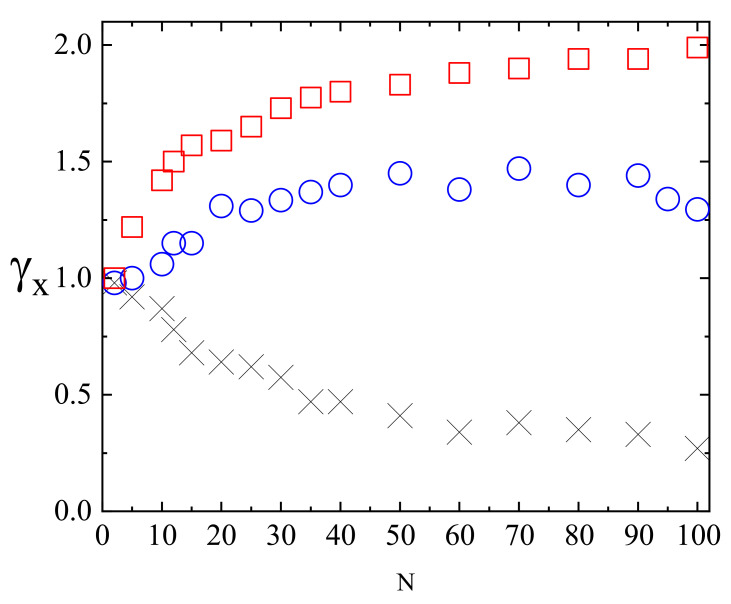
Exponent $\gamma_{x}$ as function of chain’s length *N* for the system without illumination (black crosses), homogeneous illumination with $I_{0}=0.2$ (blue circles) and inhomogeneous illumination with ∇I=0.005 (red squares). T=0.25. Based on [163].

The dynamics of a chain depends on its length (number of monomers *N*). Figure 15 shows the plots of $\gamma_{x}\left(N\right)$ for three cases: systems without illumination, with constant illumination and with both constant (offset) illumination and gradient. In the first case a sub-diffusive dynamics is present; moreover, $\gamma_{x}$ decreases as *N* increases. The plot was fitted with square-root-law $\gamma_{x}\propto N^{-1/2}$ which, interestingly, corresponds to Zimm model [4] with hydrodynamic interactions. On the contrary, in both cases with light illumination the exponent $\gamma_{x}$ becomes an increasing function of *N*. In particular, when the gradient term is present, long chains display a nearly ballistic motion. Those results indicate that collective correlations are present in the system—further studies are necessary to characterize their origin.

#### 3.4.2. 2D

**Figure 16:**
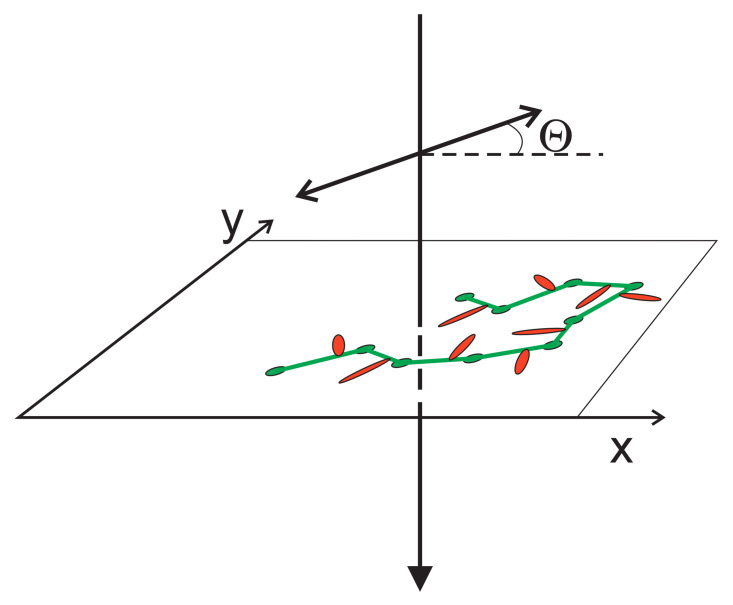
Scheme of 2D azo-dye system interacting with linearly polarized light.

Two-dimensional azo-polymer system makes possible a systematic study of the influence of light polarization on photoinduced dynamics—the topic which is technically much more demanding in three dimensions. Two limiting models of single-chain light-driven dynamics were studied in Ref. [164]: polarization-independent (PI) model and full-polarization model (FP model), see Section 2.3.4. The BFM chain and the attached azo-dye molecules lie in x−y plane while the light, linearly polarized in the same plane, propagates along *z* direction. Vector of light polarization makes an angle Θ with *x* direction, see Figure 16.

**Figure 17:**
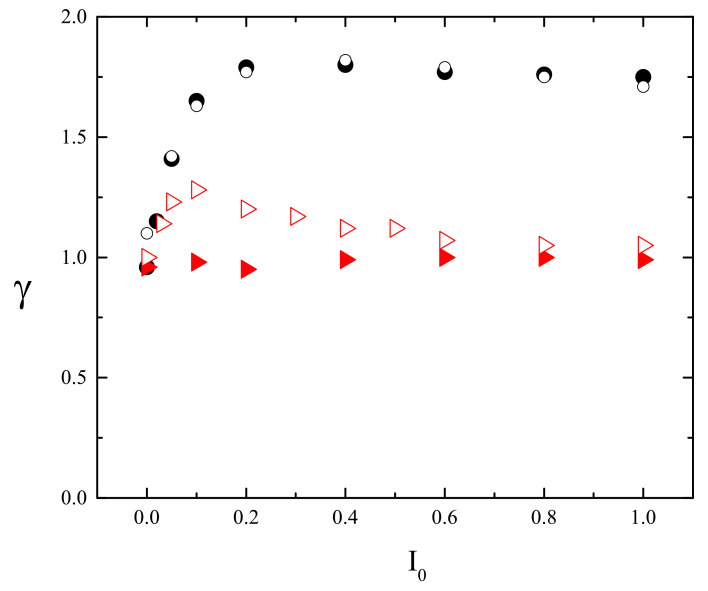
Plot of exponent $\gamma (I_{0})$. FP model: $\Theta =0^{\circ }$, ∇I=0 (black circles) and ∇I=0.005 (empty circles). PI model: ∇I=0 (red triangles) and ∇I=0.005 (empty red triangles). T=0.15.

The two models display very different types of light-driven dynamics. For constant illumination, $I\left(x\right)=I_{0}$, the PI model undergoes standard diffusion (γ=1) for $0<I_{0}<1$, while the FP model displays strongly developed super-diffusive dynamics (γ≈1.8) for $I_{0}>0.2$, see Figure 17. The effect of inhomogeneous light illumination is rather unexpected—namely, the plot of exponent $\gamma (I_{0})$ for FP model practically coincides with its counterpart for constant illumination. On the contrary, the PI model becomes moderately super-diffusive.

**Figure 18:**
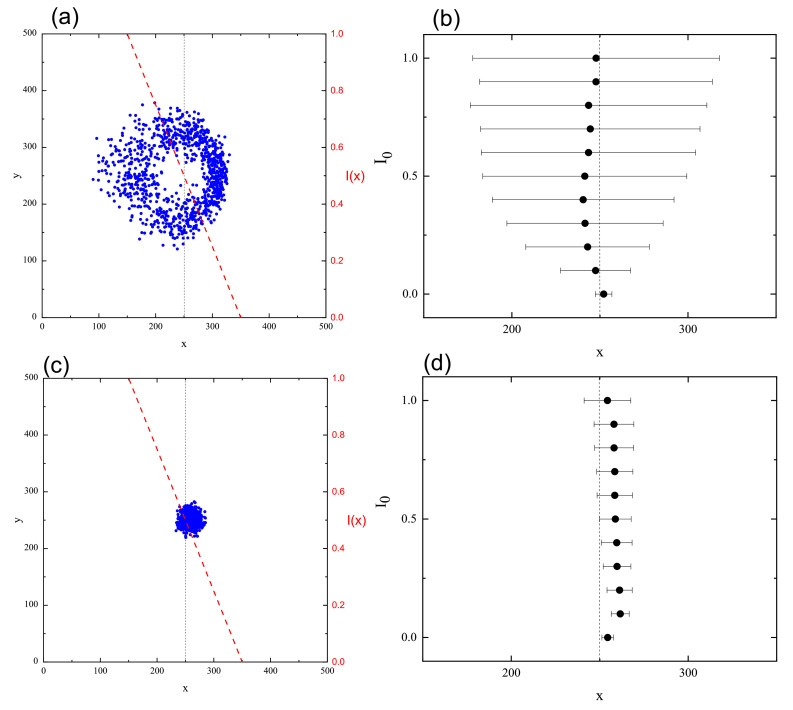
Final positions of CM of chains after $10^{4}$ MCS for FP (**a**) and PI model (**c**) and averaged position of CM in *x* direction, with standard deviation $\sigma_{x}\left(I_{0}\right)$, for FP model (**b**) and PI model (**d**). $\Theta =90^{\circ }$. T=0.15, $\nabla I=0.005,I_{0}=0.5$.

Mass transport becomes strongly dependent on the polarization in the case of inhomogeneous light illumination. Figure 18 shows the final positions of CM of the chains after $10^{4}$ MCS, as well as the average position of the CM for both models. In FP model the chains escape from their starting position and give rise to two mass currents, which move along and against the light intensity gradient. As the result, the center of the simulation cell is nearly free of the chains (Figure 18a). More importantly, an overall directed mass transport is absent—the average CM stays close to its initial position (Figure 18b). The flow of CM of chains in PI model is much more limited and, in particular, there is no empty space in the middle of the distribution of CM (Figure 18c). Contrary to the FP model case, directed photoinduced mass transport is present (Figure 18d). For longer time interval this effect becomes more pronounced.

The results presented above lead to an interesting hypothesis, which states that the origin of super-diffusive dynamics is promoted by breaking a specific symmetry present in the PI model. Namely, all the azo-dyes interact in the same way with linearly polarized light, independently on their actual localization along the chain, promoting normal diffusion. The FP model fully breaks this symmetry, leading to super-diffusion. Thus, hypothetically, a weak breaking of this symmetry brings about weak super-diffusion.

To summarize, the scheme of coupling of light polarization to azo-dye molecules plays central role in modeling, and can lead to very different dynamical regimes of photoinduced mass transport.

### 3.5. Inscription of Diffraction Gratings in Polymers and Bio-Polymers

#### 3.5.1. Polymers with Embedded Azo-Dye Molecules

**Figure 19:**
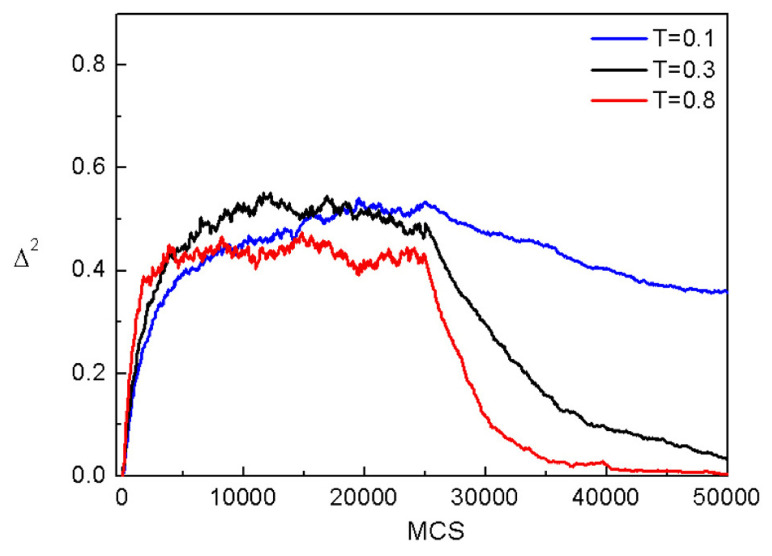
Typical MC-kinetics of diffraction efficiency $\Delta^{2}$ in the process of inscription and erasure of diffraction gratings.

The diffraction efficiency of diffraction gratings inscribed in DTWM experiment depends on temperature, type of polymer matrix and of embedded guest molecules. Their impact on the dynamics of inscription and erasure of diffraction gratings was studied for a simplified case, namely for a two-dimensional polymer matrix at temperatures below (T=0.1), close to (T=0.3) and above (T=0.8) the glass transition temperature [145]. The azo-dye molecules, located at the nodes of the 2D lattice, could reorient in 3D. A few types of kinetics of inscription and erasure were found, depending on temperature and parameters of the model: $p_{TC},R$ and $p_{diff}$. One of them is shown in Figure 19; the inscription phase ended after 2000 MCS and the erasure took place in the darkness (I=0). The diffraction efficiency weakly depends on the temperature in the inscription phase. On the contrary, the low-temperature grating is much more stable than high-temperature gratings in the erasure phase. For larger values of parameter $p_{TC}$ the grating inscribed at low temperatures is still more stable, but its diffraction efficiency is lower than that of high-temperature gratings [145].

**Figure 20:**
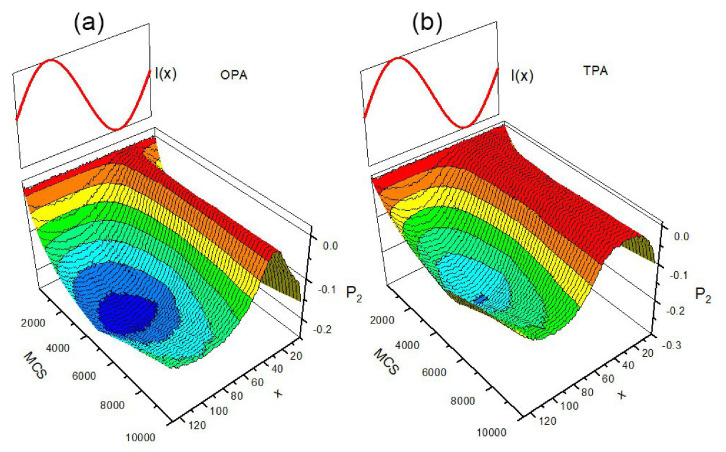
MC-time evolution of order parameter $P_{2}\left(x\right)$ for OPA (**a**) and TPA (**b**) photo-selections.

MC modeling offers some insight into “microscopic” processes leading to photo-orientation of azo-dye molecules promoted by various photo-selection rules, in particular those corresponding to one-photon (OPA) and two-photon (TPA) absorptions, see Section 2.3.4. This topic was studied in Ref. [161] using the gauge $k_{TC}^{\left(2\right)}=k_{TC}$. Figure 20 shows the kinetics of local order parameter $P_{2}\left(x\right)$ in both cases, driven by modulated light intensity and close to the glass transition temperature. Azo-dye molecules were embedded in a 3D standard polymer matrix. The profiles of order parameter are different in both cases, reflecting the differences between photo-selection rules, Equations (25) and (28): *I* vs. $I^{2}$ and $\cos^{2}\theta$ vs. $\cos^{4}\theta$. Discussion of the kinetics of concentration of *trans* molecules and of the distribution of the orientations of their long axes can be found in Ref. [145].

#### 3.5.2. Bio-Polymers: Semi-Intercalation Scenario

Experimental study of grating recording in deoxyribonucleic (DNA)-based polymers [190] showed strong differences in comparison with grating inscription in traditional host-guest systems like, e.g., PVK, PS, PMMA, Ormosil or nematic liquid crystal. First of all, the inscription under s−s polarization conditions in DNA-CTMA loaded with DR1 guest molecules was at least one order of magnitude faster then for other matrices. On the other hand, the diffraction efficiency was noticeably lower. The grating inscription followed, rather surprisingly, a single-exponential kinetics with the grating build-up time weakly dependent on the wavelength.

**Figure 21:**
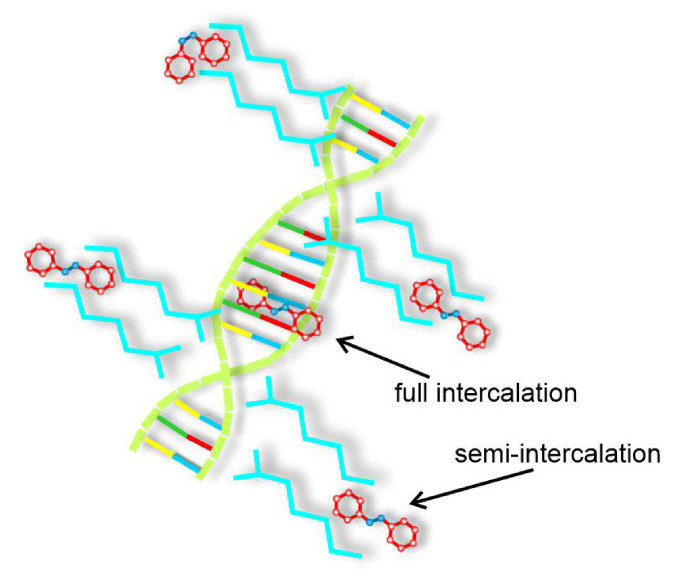
Scheme of semi-intercalation and full intercalation of an azo-dye molecule in DNA-CTMA biopolymer.

Monte-Carlo BFM modeling of those effects [155] was based on semi-intercalation scenario formulated theoretically [191,192] and experimentally [193] as a complementary mechanism to full intercalation [194] and guest-host scenario [126]. Some arguments against the full intercalation hypothesis were raised in Ref. [195]. In semi-intercalation scenario the dye can undergo photo-isomerization cycles, but the memory about the initial *trans* orientation (i.e., before trans-cis transition) is preserved. In full intercalation scenario the photo-isomerization is mechanically blocked, while in host-guest systems this memory is fully or partially lost. The scheme of semi-intercalation is shown in Figure 21.

**Figure 22:**
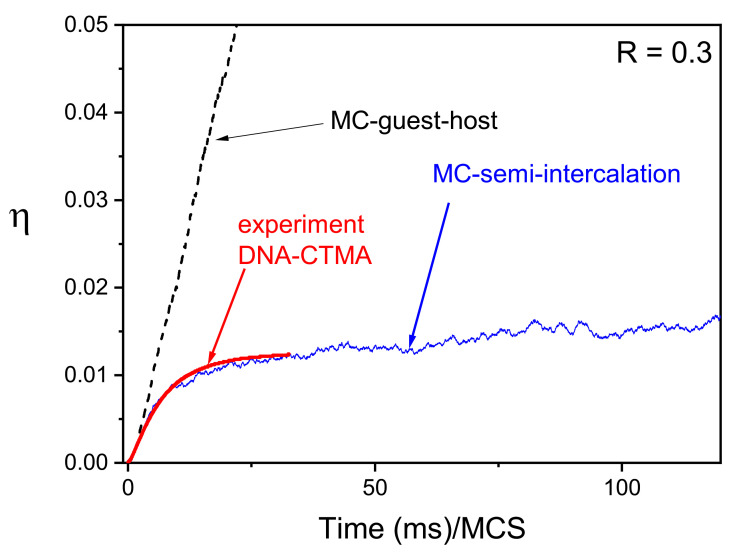
The kinetics of grating inscription, characterized by scaled diffraction efficiency $\eta =\Delta^{2}$, in DNA-CTMA-DR1 experiment and in MC semi-intercalation and host-guest systems.

A simplified Monte Carlo modeling did not account for the direct influence of the dynamics of the matrix on the azo-dyes. As the result, the model corresponds to a system of free azo-dyes with full memory about their initial *trans* orientations, with the transition rates dependent on the value of local void parameter *V*. The simulations were done for R=0.3 (Equation (27))—the value estimated on the basis of experimental data [155] for the range of those wavelengths (460nm<λ<540nm) where a weak dependence of build-up time was found experimentally. Since build-up times in semi-intercalation model depend only on the value of parameter *R*, the corresponding weak dependence was reproduced by MC simulations. Experimental data for scaled diffraction efficiency as well as the results of Monte Carlo modeling of diffraction efficiency $\eta =\Delta^{2}$ are presented in Figure 22. For comparison, MC results for host-guest system are included.

The build-up time in semi-intercalation model is much smaller than in the case of guest molecules dispersed in host matrix. On the other hand, the diffraction efficiency in the former case is much smaller than in the latter. Thus, MC modeling, having reproduced the main experimental results, offers a strong support for semi-intercalation scenario. The results indicate that biopolymeric matrices are characterized by low values of parameter *R* (e.g., R=0.3), while traditional matrices—by larger values, e.g., R=5 for polystyrene. This conclusion illustrates the effect (for a given guest molecule), namely the dependence of *R* on the matrix in which the guest molecule is dispersed.

### 3.6. SHG in Poled Polymers: Pre-Poling History Paradigm

Dipolar molecules poled with an external electric field break the centrosymmetry; the load parameter $L=N<\cos^{3}\theta >$ is directly related to SHG signal, see Equation (35). This formula predicts a linear dependence of L on number density *N*. Deviations from such dependence are well known [196,197]—saturation or a maximum of SHG susceptibility in function of *N* is observed in guest-host [198,199,200,201] chromophore—polymer systems. Various competing mechanisms, more or less pronounced in specific systems, are responsible for the departure from the linear dependence [202]. In particular, the fall-off of SHG susceptibility is ascribed to a hypothetical mechanism responsible for the aggregation of dipoles. Antiparallel pairs decrease the degree of acentric order (see Refs. [203,204] and references cited therein). Statistical mechanics and MC approaches to the problem were originated by seminal papers [205,206], and were followed by lattice [207] and off–lattice [208,209] MC simulations, Molecular Dynamics simulations [210,211], density functional theories [212], mean field theories [213,214], fully atomistic modeling [215,216,217], extended dipole models [218], or inclusion of matrix into MC simulations [219]. Those studies have formulated a few physical pictures of the poling dynamics, giving rise to various paradigms [203,204].

Another paradigm, originally formulated in Ref. [204], which ties in the maximum on the poling curve with the pre-poling history of the probe, was studied using generalized MC BFM model [156]. It was found that the physical processes in the pre-poling phase promote stretched–exponential build–up of polar phase. For this case low SHG signal and a maximum of $\chi_{ZZZ}^{\left(2\right)}$ as function of number density *N* of dipoles were observed. On the contrary, the build–up of polar phase in freshly prepared films occurs quickly, is characterized by other type of non–exponential relaxation, yields larger amplitude of SHG susceptibility and displays a saturation instead of a maximum of $\chi_{ZZZ}^{\left(2\right)}$. The origin of different kinetics is ascribed to a kind of spatial entanglement in the pre-poling interval of string-like structures containing dipoles in head–to–tail order. The details are as follows.

An off-lattice system consisting of point dipoles was embedded in a standard 3D polymer matrix, close to $T_{g}$. The potential energy included point dipole–dipole interaction, interaction of dipoles with poling field and repulsive soft–sphere interaction:

(48) $$U=\sum_{i\neq j}\frac{1}{4\;\pi \;\epsilon_{0}\epsilon }\frac{1}{r_{ij}^{3}}\left[{\vec{\mu }}_{i}\cdot {\vec{\mu }}_{j}-3\left({\vec{\mu }}_{i}\cdot {\hat{r}}_{ij}\right)\left({\vec{\mu }}_{j}\cdot {\hat{r}}_{ij}\right)\right]-\sum_{i}\vec{E}\cdot {\vec{\mu }}_{i}+\epsilon_{LJ}\sum_{i\neq j}{\left(\frac{\sigma }{r_{ij}}\right)}^{12},$$

where $\vec{E}$ denotes electric poling field, ${\vec{\mu }}_{i}$—*i*-th dipole moment, $r_{ij}$ and ${\hat{r}}_{ij}$—distance and unit vector between dipoles *i* and *j*, respectively. ϵ is the dielectric constant of the host, and σ denotes the characteristic scale for soft-sphere interactions. The following values of parameters were used: μ=26 D, σ=7 Å, $\epsilon_{LJ}/\left(k_{B}\;T\right)=0.1$, ϵ=4, E=150 V/μm, T=350 K. In the case of soft core and electrostatic interactions the cutoff was equal 5 nm. The reaction field method was also tested. Each dipole made a trial move, translational or orientational. The move was successful when it was accepted by Metropolis acceptance rule and was allowed by steric restrictions. In the initial configuration the orientations of the dipoles were random.

Of main interest was the MC kinetics of SHG susceptibility $\chi_{ZZZ}^{\left(2\right)}\propto L$. Two cases were studied. In the first one (pre-poling scenario) the first $10^{5}$ MCS constituted the pre-poling phase, with electric field switched off; the poling started after this period. In the second case the poling electric field was turned on from the very beginning.

**Figure 23:**
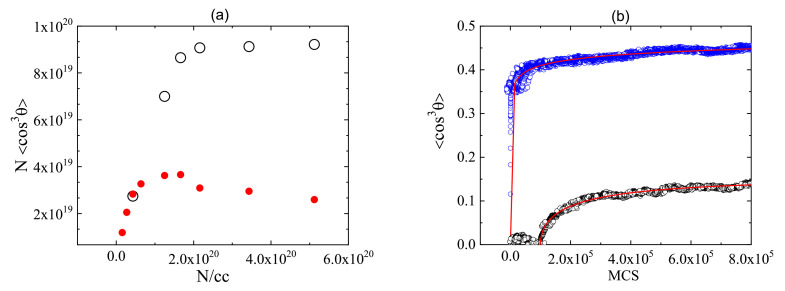
(**a**) Plot of load parameter $N\;<\cos^{3}\theta >\propto \chi_{ZZZ}^{\left(2\right)}$ as a function of number density *N*: pre-poling scenario (red) and immediate poling (black). (**b**) MC kinetics of acentric order parameter $<\cos^{3}\theta >$ for $N=2.16\times 10^{20}$/cc, for pre-poling scenario (black) and immediate poling (blue). Solid lines: stretched–exponential fits.

Figure 23a shows the plots of the load parameter $N\;<\cos^{3}\theta >$ as a function of number density *N*. The presented results speak in favor of the pre-poling history paradigm. Namely, the system with pre-poling history leads to a maximum on poling curve approximately at number density $N_{m}=1.7\times 10^{20}$/cc. On the contrary, in the case of an immediate poling a saturation takes place. The MC kinetics of acentric order parameter $A=<\cos^{3}\theta >$ is different in both cases, see Figure 23b. The system with pre-poling history remains globally isotropic (A=0) in pre-poling period; afterwards, parameter *A* smoothly grows up, reaching finally the saturation value $A_{s}\approx 0.15$. The kinetics of build–up of polar phase follows the stretched–exponential law

(49) $$<\cos^{3}\theta >\left(t\right)\propto \exp\left(-{\left(t/\tau \right)}^{\alpha }\right),$$

with α≃0.5. In the case of an immediate poling the initial rapid increase of *A* stops rather abruptly after $4\times 10^{4}$ MCS, with $A_{s}\approx 0.45$, three times higher than in the pre-poling scenario. The simulation data in a short initial time interval cannot be modeled using stretched–exponential fit.

**Figure 24:**
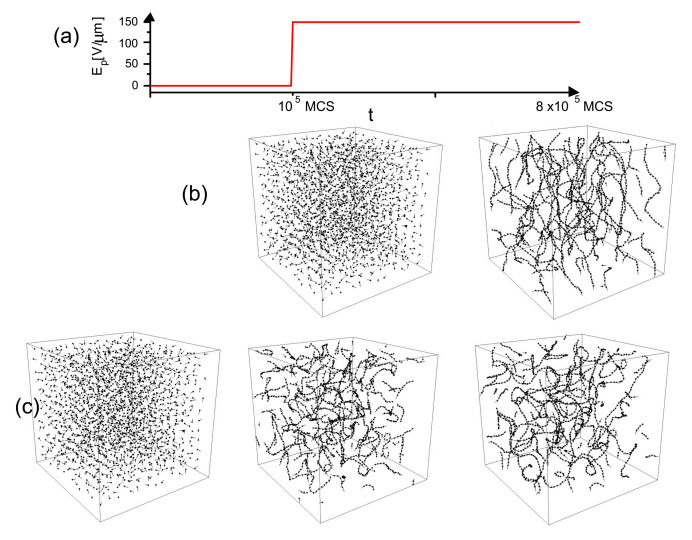
(**a**) Poling electric field as function of number of MC steps. (**b**) Configurations of dipoles: immediate poling; (**c**) pre-poling scenario. Horizontal alignment corresponds to time from top plot: t=0, $10^{5}$ MCS and $8\times 10^{5}$ MCS. Polymeric chains are not shown.

The maximal amplitude of SHG susceptibility, proportional to $L(N_{m})$, is larger in the case of immediate poling, indicating that in the pre-poling phase the dipolar component develops some kind of orientational correlations which have an impact on the poling process. Figure 24 shows chosen configurations of dipoles. In the case of direct poling the initial isotropic configuration gives rise to, after $8\times 10^{5}$ MCS, a partially ordered system of dipolar strings with a preferential order along the direction of the poling field (*z*-axis) with a dominant head-to-tail dipolar organization. Such a configuration contributes in a coherent way to acentric order parameter $<\cos^{3}\theta >$. The dynamics in the case of pre-poling scenario is different. At the end of pre-poling phase a complex spatial organization of dipolar strings is present, which displays no traces of polar order: A=0. The final configuration is clearly different than in the previous case. The dipolar strings perpendicular to the poling directions, which have their origin in the configuration at the end of pre-poling phase, do not contribute to the acentric order parameter, leading to lower values of SHG susceptibilities.

Crude estimations lead to the conclusion that important reorganization of dipolar structure close to the glass transition temperature can take place within a few minutes [156].

### 3.7. All Optical Poling

In all-optical poling experimental technique two linearly polarized light beams with frequencies ω and 2ω interact with embedded azo-dye molecules and promote polar order [162], necessary for SHG effect (Section 2.4.2).

A modified MC BFM model was used to study the influence of glass transition onto kinetics of guest molecules in all-optical poling and, in particular, to characterize the “transfer” of complexity from the matrix onto the guest molecules [150]. The main conclusion is that the dynamics of azo molecules close to $T_{g}$ is complex: it exhibits power-laws and a non-exponential relaxation. The details are as follows.

Noninteracting photoswitchable model azo-dye molecules were dispersed in BFM matrix. Their concentration was low and, correspondingly, their impact on the dynamics of the matrix was neglected. The temperature of glass transition $T_{g}$ was estimated as $0.23<T_{g}<0.26$. The transition rate $p_{T}\left(\theta \right)$ is given by Equation (29). Here, θ denotes an angle which the long axis of *trans* molecule makes with the vector of linearly polarized light beams.

**Figure 25:**
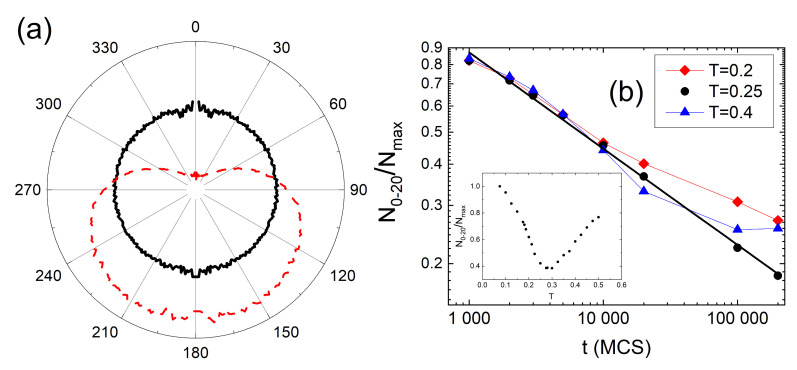
(**a**) Polar plot of angular distribution ρ(θ)/sinθ for t=0 (solid black line) and $t=2\times 10^{5}$ MCS (dashed red line). (**b**) Double logarithmic plot of $N_{0-20}\left(t\right)/N_{max}$ for T=0.2,0.25 and 0.4. Inset: temperature dependence of normalized parameter $N_{0-20}/N_{max}$ for $t=2\times 10^{5}$ MCS. See text for more details. Based on [150].

The distribution of orientations of *trans* molecules is characterized by time-dependent probability density ρ(θ,t). The interpretation of the data becomes more clear for polar plots of function ρ(θ,t)/sinθ. Initially, the distribution of the orientations is isotropic (black circle, Figure 25a). In the process of poling the concentration of molecules with $\theta =180^{\circ }$ increases at the cost of the decrease of the concentration of those with $\theta =0^{\circ }$ (angular hole burning (AHB) effect), promoting polar order (dashed red line). This effect is quantified using normalized function $N_{0-20}\left(t\right)/N_{max}$, where $N_{0-20}\left(t\right)$ denotes the number of molecules in trans state with $0^{\circ }\leq \theta \leq 20^{\circ }$ and $N_{max}$ is a constant. Double logarithmic plot of $N_{0-20}\left(t\right)/N_{max}$, shown in Figure 25b, is linear for T=0.25, corresponding to power law

(50) $$N_{0-20}\left(t\right)∼t^{\alpha },\;\;\alpha \approx 0.3.$$

For temperatures slightly lower (T=0.2) and higher (T=0.4) power law is no more valid. The magnitude of AHB effect depends on the temperature; it is the strongest in the temperature interval 0.275<T<0.3, see the inset in Figure 25b, which shows the temperature-dependence of parameter $N_{0-20}\left(t\right)/N_{max}$ for $t=2\times 10^{5}$ MCS (end of simulation). 

**Figure 26:**
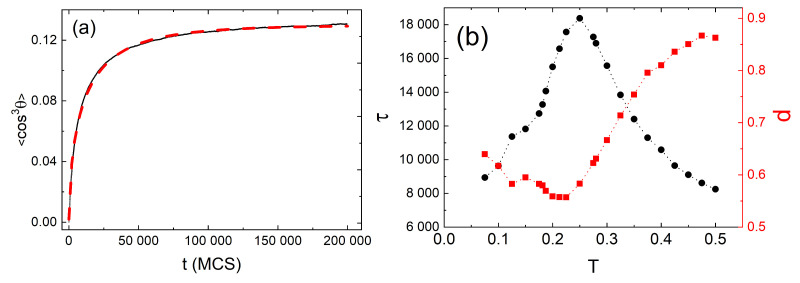
(**a**) MC-time dependence of acentric order parameter $\langle \cos^{3}\theta \rangle \left(t\right)$ for T=0.15 (solid black line). Dashed red line represents stretched exponential fit, Equation (51). (**b**) Temperature dependence of parameters of the fits: τ(T) (left axis) and d(T) (right axis).

Nonlinear SHG susceptibilities are directly proportional to accentric order parameter $\langle \cos^{3}\theta \rangle$ (Equations (33) and (34)), because the number density *N* of azo-dyes in *trans* state was practically constant in the process. This order parameter can be calculated directly from MC simulations or using probability density ρ(θ,t). An exemplary plot of its time-dependence is shown in Figure 26a. It was found that stretched exponential fit represents, in contrast to other fits used, a statistical model in a wide interval of temperatures below and above glass transition temperature:

(51) $$\langle \cos^{3}\theta \rangle \left(t\right)=A\left[1-e^{-{\left(t/\tau \right)}^{d}}\right].$$

Parameters of the fit, exponent *d* and characteristic time τ, depend on the temperature, see Figure 26b. Characteristic time displays the slowing-down effect with maximum around T=0.25. Exponent *d* has values well below d=1, which corresponds to one-exponential kinetic; its minimum (d≈0.55) is located around T=0.22.

Those results represent the effect of “transfer” of complex behaviour from polymer matrix onto guest molecules, most evident in close vicinity of the glass temperature. A detailed discussion of this effect, as well as its relation to the dynamics of *mosaic-like* states, can be found in the original paper [150].

### 3.8. Photomechanical Effect in Polymeric Fibers

The BFM model of host-guest systems is open to generalizations. In this section we modify it [153] to support the qualitative hypothesis [220] of the cooperative release of stress in dye-doped polymer optical fibers, directly related to the photomechanical effect.

The photomechanical effect in dye-doped polymer optical fibers was studied using experimental, theoretical and numerical approaches [80,81,221]. Nevertheless, its microscopic origin still remains unclear. A simple but sound model for an optically activated cantilever, which accounts for photothermal and photo-reorientation mechanisms [220], is based on the following hypothesis. The type of the dynamics of the photoswitchable molecules, promoted by photodriven *trans-cis-trans* isomerization cycles, depends on local characteristics of the polymer matrix, like size, shape, spatial distribution and elastic properties [222] of the voids. Those factors determine the ability of azo-dye molecules to change their orientation in the space. The interactions between the matrix and the dyes lead to strains and stresses which can, hypothetically, relax in a cooperative way, resulting in a cooperative configuration of some amount of the dyes oriented perpendicularly to the direction of the polarization of the light. Authors speculate that this effect promotes an elongation of the polymer matrix along the direction perpendicular to the vector of light polarization [220]. Similar scenario was observed in orientational approach [102,114], which predicts contraction along the polarization direction and the elongation in perpendicular direction.

**Figure 27:**
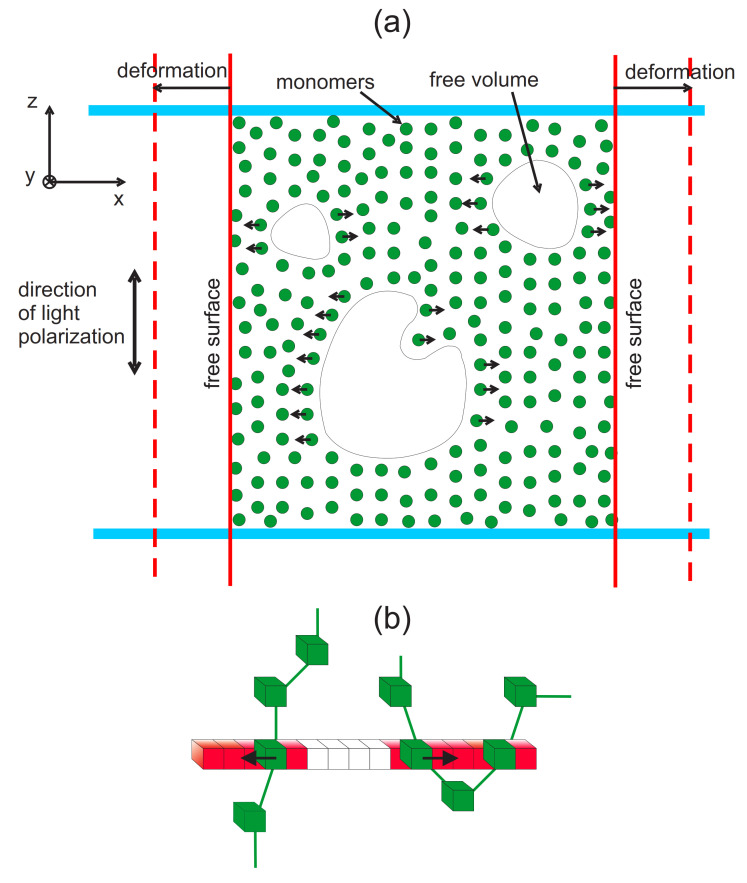
Photomechanical effect: (**a**) The model. (**b**) Scheme of stress-driven trial movements of the monomers close to the void.

The goal of the generalized BFM model [153] was to provide arguments in favour of this hypothesis. The 2D section of the model with free boundaries is shown in Figure 27a. Light, linearly polarized along *z* axis, propagates along *x* axis. The monomer-free areas schematically represent large voids (V>5) with dyes in *trans* state, oriented along *x* axis. Locally ordered dyes sustain pressure on the polymer matrix [220], and push the monomers in a close neighborhood of the void away from their positions, as shown in the figure. This effect is modeled in the following kinetic way. If the monomer is located no more than three lattice sides to the left (right) of the free volume then it performs the trial movement to the left (right), see Figure 27b.

Macroscopic deformation of the host-guest system is the net result of the stress-driven dynamics of monomers close to voids with V>5. It is characterized by the distance Δx—the displacement of the free surfaces, see Figure 27. We point out that in real systems free surfaces occur because of the surface tension, which is absent in BFM model. In the simulations the surface was stabilized by the surface-tension like force [187], see Section 3.3.

**Figure 28:**
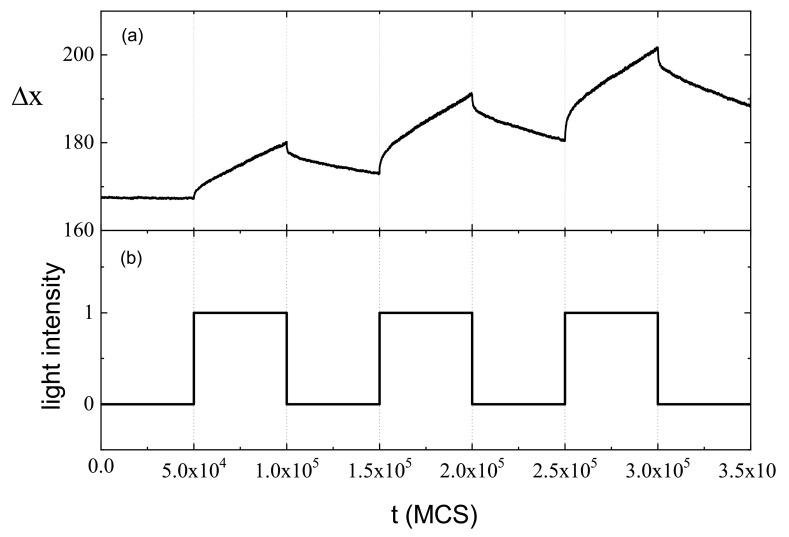
Plot of elongation Δx of the host–guest system in function of MC time (**a**), driven by periodic modulation of light intensity (**b**).

Figure 28a shows the plot of the elongation Δx of the system in the presence of periodically modulated light intensity (on and off phases). In the bright phase (light on) the system expands, while in the dark period (light off) the dyes in trans state undergo rotational diffusion, thus removing the source of stress acting on neighbouring monomers. As the result, the system shrinks. To summarize, the model supports the hypothesis formulated in Ref. [220] and constitutes a reliable starting point for more detailed studies of photomechanical effects.

### 3.9. Continuous Time Random Walk and Toy Model of SRG Inscription

Monte Carlo study of the dynamics of an azo-dye system under modulated light illumination has established the physical picture of the chain dynamics which accompanies the process of SRG inscription as corresponding to a subtle dynamical coexistence of sub-diffusion, normal diffusion and super-diffusion in separate parts of the system. The driving parameter is a local value of light illumination, see Section 3.4 and Ref. [163].

This scenario offers the possibility of a simple (“toy”) modeling of this process, based on mathematical formalism of Continuous Time Random Walk (CTRW) [223], which constitutes a generalization of a standard diffusion model. Two parameters are introduced which control the jumps in space and in time. The former is distributed according to Levy α-stable distribution. The waiting times between the jumps are distributed according to one parameter (β) Mittag-Leffler distribution. The squared displacement displays power-law behaviour [223]:

(52) $${\left(\Delta r\right)}^{2}\left(t\right)\propto t^{-\delta },\;\delta =\frac{2\beta }{\alpha }.$$

**Figure 29:**
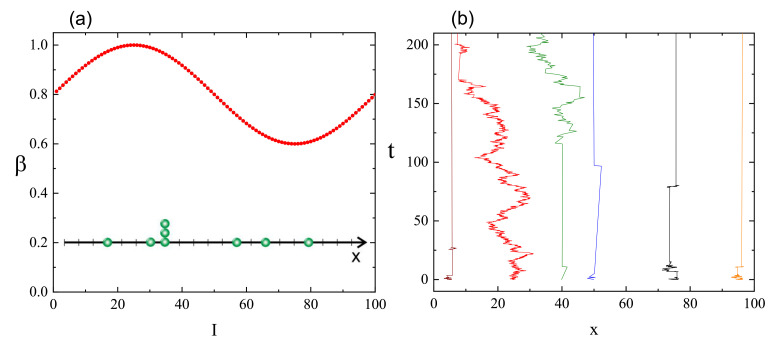
CTRW model in 1D. (**a**) Spatial modulation of parameter β. Inset: independent walkers on a line. (**b**) Exemplary trajectories of walkers, α=2.

The toy model [163] of an azo-polymer system in the presence of an inhomogeneous light illumination is a set of non-interacting, independent point walkers, which represent the centers of mass of polymer chains. The walkers perform CTRW on a line (Figure 29), with α=2 corresponding to gaussian distribution of lengths of jumps along the line. Correspondingly, δ=β and the type of dynamics depends on the value of parameter β: it is sub-diffusive for β<1, super-diffusive for β>1 and corresponds to normal diffusion for β=1. This classification is the same as its counterpart using parameter γ, Equation (37). Thus, in CTRW model exponent γ has analytical representation: it is identified with β:

(53) γ=β.

**Figure 30:**
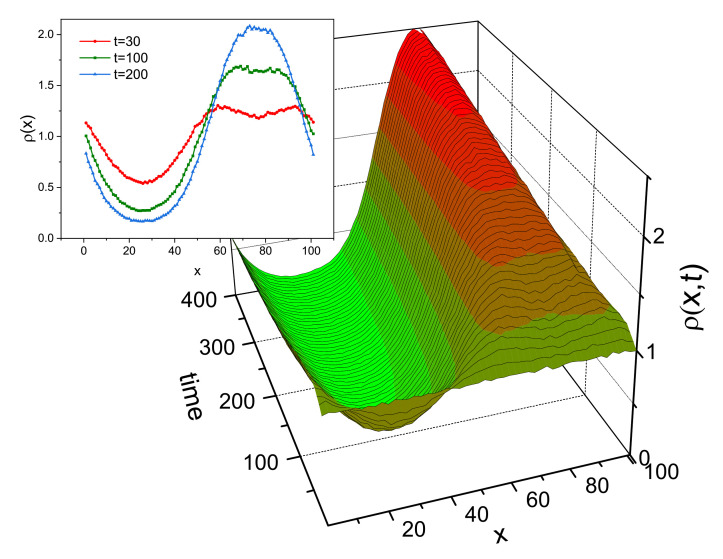
Plot of density ρ(x,t) of walkers in CTRW model. α=2.

While the dependence of β on *x* can be found from relations γ(I) (Figure 12) and formula I(x) Equation (20), the model used a simplified version, namely a sinusoidal modulation of β (Figure 29). CTRW was simulated using the method of Ref. [224]. The trajectories of the walkers shown in Figure 29 display a variety of behaviours, from oriented motion through diffusive motion to dynamical arrest. Various types of motion contribute to the time-dependent density ρ(x,t) of walkers, shown in Figure 30. This plot is typical for MC simulations of density of chains in the process of inscription of SRG as discussed in Section 3.3. Moreover, the fine structure of the grating appears as a transient effect, corresponding to one of the scenarios introduced in Ref. [154] and discussed in Section 3.3. To summarize, a rather naive model based on the concept of CTRW offers a promising starting point for analytical studies of light-driven mass transport in azo-polymers.

## 4. Conclusions

Generalized Monte Carlo bond fluctuation models of host-guest systems together with theoretical formalism presented in this review demonstrate great capabilities to describe the photo-orientation and photo-deformation of broad classes of azobenzene-containing polymers, like, e.g., polymer melts, solutions, brushes, dendrimers, liquid crystalline polymers and others.

Simulation Monte Carlo methods offer, on the one hand, the possibility of both qualitative and quantitative predictions and theoretical engineering for host-guest systems, in the context of their non-linear optics applications. On the other hand, this methodology provides tools promoting a deeper understanding of various non-linear optical processes in polymeric systems and it consolidates the physical picture of host-guest systems as complex ones. As the side effect, the host-guest systems constitute a kind of a theoretical lab which offers some intuition about the origin of complexity in physical models. The characterization of “microscopic” mechanisms leading to semi-macroscopic complex diffusion of the azo-polymer chains constitutes a challenge in the area of non-linear dynamics of chain-like objects. This topic is under study now; the results will be published elsewhere.

The theoretical formalism allows to calculate the light-induced mechanical stress from the kinetic equations of photoisomerization. According to the theoretical calculations, the mechanical stress can achieve the order of magnitude of several GPa at conventional light intensities ∼100 ${\mathrm{mW}/\mathrm{cm}}^{2}$. Similar characteristic values of the light-induced mechanical stress were estimated in the experimental studies [111,112,113], which show that the light-induced force is able to deform chemical bonds and to break metallic layers deposited on the surface of a glassy azo-polymer. Thus, the proposed theoretical formalism provides an explanation of the photodeformation phenomena from the first principles. In particular, the experimental results mentioned above can be explained avoiding additional assumptions, such as the photofluidization hypothesis, which fails for the azo-polymers deep in the glassy state.

Furthermore, the presented theoretical approach is able to predict not only the magnitude of the photodeformation but also its direction with respect to the polarization vector of the light, depending on the chemical structure of azo-polymers. It is shown in the review that the theoretical formalism developed earlier for photodeformation and photo-ordering under the linearly polarized [114,115,116,117,118,132,133,134,135,136,137,138] or circularly polarized [119] light can be generalized to study these phenomena under the elliptically polarized light illumination. It opens up the possibility to investigate in further studies the phenomena of photodeformation and photo-ordering in azo-polymers under illumination with the light of arbitrary polarization using both theoretical methods and computer simulation techniques.

Simulation methods discussed in this review can be applied to modeling of a wider class of physical processes in polymer matrices in the presence of light illumination like, e.g., developing of polymer-based materials by UV-Vis and gamma radiations, using UV cross linking [225,226,227], gamma sterilization [228], gamma functionalization [229] and surface modification [230]. Another interesting area of applications is related to the effects of photo-ordering in azo-polymers irradiated with high-energy ions. The structural damages of the host-guest system modify the local free volume characteristics and, in consequence, the amplitude of various non-linear effects. The interaction of the ions with the system can be modeled using the dedicated software GEANT4 [231,232,233]. 

## Data Availability

The data presented in this study are available on request from the corresponding author.
